# DZNep promotes mouse bone defect healing via enhancing both osteogenesis and osteoclastogenesis

**DOI:** 10.1186/s13287-021-02670-6

**Published:** 2021-12-20

**Authors:** Xiankun Cao, Wenxin He, Kewei Rong, Shenggui Xu, Zhiqian Chen, Yuwei Liang, Shuai Han, Yifan Zhou, Xiao Yang, Hui Ma, An Qin, Jie Zhao

**Affiliations:** 1 Shanghai Key Laboratory of Orthopedic Implants, Department of Orthopaedics Surgery, Shanghai Ninth People’s Hospital, Shanghai Jiao Tong University School of Medicine, No. 639, Zhizaoju Road, Shanghai, 200011 People’s Republic of China; 2 Department of Orthopaedics, Mindong Hospital Affiliated to Fujian Medical University, Fuan, 355000 Fujian Province People’s Republic of China; 3 Department of Orthopedics, Sun Yat-Sen Memorial Hospital, Sun Yat-Sen University, Guangzhou, 510120 People’s Republic of China; 4 Guangxi Key Laboratory of Regenerative Medicine, Guangxi Collaborative Innovation Center for Biomedicine, GuangxiASEAN Collaborative Innovation Center for Major Disease Prevention and Treatment, Guangxi Medical University, Nanning, 530021 Guangxi People’s Republic of China

**Keywords:** DZNep, Osteoclast, Osteoblast, Bone defect, EZH2-H3K27me3-Wnt signaling pathway, EZH2-H3K27me3-Foxc1-NF-κB signaling pathway

## Abstract

**Background:**

Enhancer of zeste homolog 2 (EZH2) is a novel oncogene that can specifically trimethylate the histone H3 lysine 27 (H3K27me3) to transcriptionally inhibit the expression of downstream tumor-suppressing genes. As a small molecular inhibitor of EZH2, 3-Deazaneplanocin (DZNep) has been widely studied due to the role of tumor suppression. With the roles of epigenetic regulation of bone cells emerged in past decades, the property and molecular mechanism of DZNep on enhancing osteogenesis had been reported and attracted a great deal of attention recently. This study aims to elucidate the role of DZNep on EZH2-H3K27me3 axis and downstream factors during both osteoclasts and osteoblasts formation and the therapeutic possibility of DZNep on bone defect healing.

**Methods:**

Bone marrow-derived macrophages (BMMs) cells were cultured, and their responsiveness to DZNep was evaluated by cell counting kit-8, TRAP staining assay, bone resorption assay, podosome actin belt. Bone marrow-derived mesenchymal stem cells (BMSC) were cultured and their responsiveness to DZNep was evaluated by cell counting kit-8, ALP and AR staining assay. The expression of nuclear factor-κB (NF-κB), mitogen-activated protein kinase (MAPK), Wnt signaling pathway was determined by qPCR and western blotting. Mouse bone defect models were created, rescued by DZNep injection, and the effectiveness was evaluated by X-ray and micro-CT and histological staining.

**Results:**

Consistent with the previous study that DZNep enhances osteogenesis via Wnt family member 1(Wnt1), Wnt6, and Wnt10a, our results showed that DZNep also promotes osteoblasts differentiation and mineralization through the EZH2-H3K27me3-Wnt4 axis. Furthermore, we identified that DZNep promoted the receptor activator of nuclear factor-κB (NF-κB) ligand (RANKL)-induced osteoclast formation via facilitating the phosphorylation of IKKα/β, IκB, and subsequently NF-κB nuclear translocation, which credit to the EZH2-H3K27me3-Foxc1 axis. More importantly, the enhanced osteogenesis and osteoclastogenesis result in accelerated mice bone defect healing in vivo.

**Conclusion:**

DZNep targeting EZH2-H3K27me3 axis facilitated the healing of mice bone defect via simultaneously enhancing osteoclastic bone resorption and promoting osteoblastic bone formation.

**Supplementary Information:**

The online version contains supplementary material available at 10.1186/s13287-021-02670-6.

## Background

EZH2 is a novel oncogene that emerges in recent years. Due to its histone methyltransferase activity, EZH2 can specifically trimethylate the histone H3 lysine 27 (H3K27me3) to inhibit the expression of downstream tumor-suppressive genes at the transcriptional level, which promote the proliferation and decrease the apoptosis of cancer cells [1–3]. With the roles of epigenetic regulation of bone cells emerged in past decades, the function of EZH2 in bone homeostasis has been attracting a great deal of attention recently.

Bone, a dynamic tissue, constantly undergoes the procedure of new bone formation and old bone elimination [4]. Under physiological conditions, this kind of process is inseparably balanced and coordinated by the bone-resorbing osteoclasts and bone-forming osteoblasts [5, 6]. Once pathological damage such as defect and fracture occurs, both osteogenesis and subsequent osteoclastogenesis are initiated to facilitate bone healing [7, 8]. There are several precedent studies with divergence but demonstrating the importance of EZH2-H3K27me3 axis in osteoclast differentiation and mesenchymal stem cells (MSCs). Fang and colleagues reported that EZH2 was recruited to the IRF8 promoter regions after RANKL stimulation to deposit H3K27me3 and downregulate IRF8 expression and thus exerted a positive effect on osteoclastogenesis [9]. Adamik and colleagues further showed that RANKL triggers EZH2 translocation into the nucleus where it represses negative regulators of osteoclastogenesis such as MafB, Irf8, and Arg1 [10]. However, one study indicated that the inhibition of EZH2-H3K27me3 axis resulted in the transcriptional upregulation of Foxc1 [11]. Foxc1 had been verified to induce the nuclear translocation of NF-κB through promoting the phosphorylation of IκB both in mice airway smooth muscle cells and human basal-like breast cancer cells [12, 13] and the PI3K/AKT signaling pathway in ovalbumin-induced asthmatic mice [14]. Moreover, the nuclear translocation of activated NF-κB was widely acknowledged to upregulate the key downstream regulators like the nuclear factor of activated T cells c1 (NFATc1) [15] and c-Fos [16], which plays important roles in osteoclast formation [17]. Similarly in MSCs, Hemming and colleagues generated mesenchymal cell-specific conditional knock-out mice of EZH2 gene using Prx-cre drive and demonstrated that Ezh2 ± and −/− mice exhibited thinner cortical bone and decreased mechanical strength compared to the wild-type control [18]. While Jing H and colleagues reported that DZNep, a small molecular compound universally known as an inhibitor of EZH2 [2, 19, 20], shifts the differentiation of MSCs from adipocytes to osteoblasts by removing the inhibitory effect of EZH2-H3K27me3 axis on the Wnt signaling pathway [21]. To sum up, the role of EZH2-H3K27me3 axis on bone homeostasis is still controversy and the explicit mechanism remains to be further explored.

DZNep was extensively studied because of its suppressive roles on EZH2 in tumors, such as malignant peripheral nerve sheath tumor, prostate cancer, head and neck squamous cell carcinoma, gastric cancer, and acute myeloid leukemia (AML) [22–26]. As previous mentioned, the enhancing osteogenesis property of DZNep via EZH2-H3K27me3-Wnt axis had been reported. Nevertheless, there is few research discussing the role of DZNep on osteoclasts. Whether and how the EZH2-H3K27me3 axis plays an important role during osteoclastogenesis after DZNep treatment is still absence currently. Moreover, drugs and compounds regulate the formation of osteoclasts [27] and osteoblasts [28] have been widely studied for treatment of the bone defect and fracture. Exploring the potential therapeutic effect of DZNep on bone defect via regulating both osteogenesis and osteoclastogenesis in vivo also has great clinical significance.

Taken together, this study aims to elucidate the role of DZNep on EZH2-H3K27me3 axis and downstream factors during both osteoclasts and osteoblasts formation and the therapeutic possibility of DZNep on bone defect healing.

## Methods

### Reagents and antibodies

The S-adenosine homocysteine and EZH2 inhibitor DZNep were purchased from Ape × Bio (Houston, TX, USA) and were dissolved in phosphate buffer saline (PBS) as a stock solution at the concentration of 5 mM. Fetal bovine serum (FBS) was bought from Gibco BRL (Sydney, Australia), minimal essential medium alpha (α-MEM) was bought from Hyclone (Logan, UT, USA), and penicillin/streptomycin was purchased from Gibco BRL (Gaithersburg, MD, USA). Recombinant mouse RANKL and M-CSF were obtained from R&D (Minneapolis, MN, USA). The cell counting kit-8 (CCK-8) was purchased from Dojindo Molecular Technology (Japan). The TRAP staining kits were bought from Sigma-Aldrich (St. Louis, MO, USA). The SYBR® Premix Ex Taq™ II and Prime Script RT reagent Kit were obtained from Takara Biotechnology (Otsu, Shiga, Japan). The primary antibodies against β-actin, LaminB, ERK, phospho-ERK (Tr202/Tyr204), JNK, phospho-JNK (Tr183/Tyr185), p38, phospho-p38, IKKβ, IκBα, P65, phospho-IKKα/β, phospho-IκBα, phospho-P65, EZH2, H3K27me3, GSK-3β, phospho-GSK-3β(ser9), and the secondary antibody, were bought from Cell Signaling Technology (CST, Danvers, MA, USA). The primary antibodies against NFATc1, c-FOS were purchased from Absin Bioscience Inc (Shanghai, China). The primary antibodies against Foxc1, Wnt4 were purchased from Abcam (Cambridge, UK). Except β-actin is mouse anti-mouse. All the antibody mentioned are rabbit anti-mouse.

### Bone marrow-derived macrophages (BMMs) preparation and cytotoxicity assay

Primary BMMs were obtained from whole bone marrow of male 6-week-old C57BL/6 mice. In brief, cells were extracted from the tibiae and femurs bone marrow and suspended in complete α-MEM (α-MEM supplemented with 30 ng/ml M-CSF, 10% FBS, and 1% penicillin/streptomycin). Then, the cell cultures were kept in a humid environment at 37 °C with 5% CO2 until they reached 90% confluence. The cytotoxic effects of DZNep on BMMs were then determined by the CCK-8 kit. Specifically, the BMMs were seeded into 96-well plates in triplicate at the density of 8 × 103 cells/well in presence of 100μL complete α-MEM for 24 h. After that, the cells were treated with increasing concentration of DZNep (control, 3.125, 6.25, 12.5, 25, 50, 100, 200 nM) for 24, 48, and 96 h. After treatment, 100μL medium contained 10% CCK-8 buffer was added to the wells and incubated in the dark at 37 °C for 2 h. The absorbance was then detected at 450 nm wavelength (650 nm reference) on a microplate reader.

### Osteoclast differentiation and TRAP staining assay

To evaluate the role of DZNep on osteoclastogenesis, BMMs were seeded into, in triplicate, a 96-well plate at the density of 8 × 103 cells/well with complete α-MEM for one day. Afterward, the culture medium was replaced by the complete α-MEM containing different concentrations of DZNep (control, 3.125, 6.25, 12.5, and 25 nM) and RANKL (50 ng/ml) to stimulate osteoclast differentiation. Cells untreated with DZNep were included as the control group. Moreover, the BMMs treated with or without DZNep for various days were also analyzed. For EZH2 gene knockdown studies, the cells were transfected with virus constructed of different EZH2 short hairpin RNA (shRNA) and negative control shRNA by mixed with polybrene (final concentration of 10ug/ml) for 48 h. The transfection mixture was then replaced by complete α-MEM added with puromycin for 24 h, and BMMs were then seeded into a 96-well plate at the density of 1 × 104 cells/well with complete α-MEM. The culture medium was then replaced every 2 days until the matured osteoclasts could be observed in the plate. For Foxc1 inhibition experiment, the cells were separately transfected with negative control siRNA, negative control siRNA with DZNep, and Foxc1 small interfering RNA (siRNA) with DZNep and by mixed with transfection reagent and osteoclast culture medium. The transfection mixture was then replaced every 2 days until the matured osteoclasts could be observed in the plate. The plate was then washed by PBS 1 times and fixed with 4% paraformaldehyde for 15 min. TRAP staining was performed immediately without light at 37 °C for 30 min. An optical microscope (Olympus, Tokyo, Japan) was used to image the photographs, and the TRAP-positive cells with more than three nuclei were considered osteoclasts and were quantified the area and number using the ImageJ software (NIH, Bethesda, MD, USA).

### Bone resorption assay

To explore the effect of DZNep on the function of osteoclast, BMMs on complete α-MEM were seeded at the density of 1 × 104 cells/well, in triplicate, on the Osteo Assay Surface plates (Corning, NY, USA). After thirty-six hours, the culture medium was replaced by complete α-MEM, with RANKL (50 ng/mL), and various doses of DZNep (control, 6.25, 12.5, and 25 nM). For EZH2 gene knockdown studies, the cells were transfected with virus constructed of different EZH2 short hairpin RNA (shRNA) and negative control shRNA by mixed with polybrene (final concentration of 10ug/ml) for 48 h. The transfection mixture was then replaced by complete α-MEM added with puromycin for 24 h, and the survived BMMs were seeded into a 96-well plate, in triplicate, at a density of 1 × 104 cells/well. The medium needs to be constantly replaced until the osteoclast had matured for 1 day. For Foxc1 inhibition experiment, the cells were separately transfected with negative control siRNA, negative control siRNA with DZNep, and Foxc1 small interfering RNA (siRNA) with DZNep and by mixed with transfection reagent and osteoclast culture medium. The transfection mixture was then replaced every 2 days until the osteoclast had been observed for 2 days. The 5% sodium hypochlorite was used to wash the wells for 3 min to remove the cells. The total area of resorption was photographed and then calculated by ImageJ software (NIH, Bethesda, MD, USA).

### Immunofluorescence of podosome actin belt

BMMs in complete α-MEM with or without 25 nM DZNep were seeded in 6-well plates at a density of 1 × 105 cells/well and cultured for 6 days. Once the matured osteoclast could be observed, the plate was washed by PBS and then fixed with 4% paraformaldehyde. The 10 min 0.2% Triton X-PBS was then performed for permeabilizing. After three times washing by PBS, FITC-labeled phalloidin was added for 1 h in darkness to bounds with the cells F-actin ring. The nuclei were stained at RT for 5 min without light by 4′, 6-diamidino-2-phenylindole (DAPI). After the final PBS wash, the perimeter of the actin ring could be observed by the fluorescence microscope (Leica) and analyzed using ImageJ software (NIH, Bethesda, MD, USA).

### Cultivation, differentiation, and mineralization of osteoblasts

Primary BMSC were obtained from the whole bone marrow of male 4-week-old C57BL/6 mice. In brief, cells were extracted from the tibiae and femurs bone marrow and suspended in α-MEM supple with 10% FBS, and 1% penicillin/streptomycin. Then, the cell cultures were kept in a humid environment at 37 °C with 5% CO2 until they reached 90% confluence. For further identifying the effect of DZNep on osteogenesis, bone marrow-derived mouse mesenchymal stem cells were firstly seeded into the 48-well plates for one day at a density of 5 × 104 cells/well. Then the medium was changed by low-glucose DMEM with 15% FBS, 5 mM β-glycerophosphate, 50 µg/mL ascorbic acid, 10-7 mM dexamethasone, and various concentrations of DZNep (0, 12.5, 25, 50, and 100 nM). The medium was changed twice a week. For EZH2 gene knockdown studies, the cells were transfected with virus constructed of different EZH2 short hairpin RNA (shRNA) and negative control shRNA by mixed with polybrene (final concentration of 10ug/ml) for 48 h. The transfection mixture was then replaced by complete α-MEM added with puromycin for 24 h, and BMSCs were then seeded into a 48-well plate at the density of 5 × 104 cells/well with complete α-MEM for 24 h. The culture medium was then replaced by low-glucose DMEM with 15% FBS, 5 mM β-glycerophosphate, 50 µg/mL ascorbic acid, 10-7 mM dexamethasone. The medium was changed twice a week. For Wnt4 inhibition experiment, the cells were separately transfected with negative control siRNA, negative control siRNA with DZNep, and Wnt4 small interfering RNA (siRNA) with DZNep and by mixed with transfection reagent and osteoblast culture medium. The transfection mixture was then replaced every 3 days. On day 7 and day 28, cells were stained by using BCIP/NBT kit (Beyotime, Shanghai, China) to observe the percent of alkaline phosphatase (ALP)-positive cells and 1% Alizarin red S solution (Solarbio, Beijing, China) to visualize the extracellular matrix calcium deposition.

### Quantitative real-time PCR analysis

BMMs were cultured in 6-well plates at the density of 2 × 105 cells/well in α-MEM supplemented with M-CSF (30 ng/ml), RANKL (50 ng/ml). The cells were then treated with or without 25 nM DZNep for 0, 1, 3, 5 days, or with various concentrations of DZNEP (0, 6.25, 25 nM) for 5 days, or with negative control siRNA, negative control siRNA with DZNep, and Foxc1 small interfering RNA (siRNA) with DZNep for 5 days. Once the culture completed, the Axygen RNA Miniprep Kit (Axygen, Union City, CA, USA) was used to extract the total RNA. BMSC were seeded in a 6-well plate at a number of 4 × 105 cells/well and treated with 0, 25, 50, 100 nM of DZNep combined with osteoblast-cultured medium for 24 h, or with or without 100 nM DZNep combined with osteoblast-cultured medium for 0 and 7 days, or with negative control siRNA, negative control siRNA with DZNep, and Wnt4 small interfering RNA (siRNA) with DZNep combined with osteoblast-cultured medium for 7 days. Once the culture completed, the Axygen RNA Miniprep Kit (Axygen, Union City, CA, USA) was used to extract the total RNA. After reverse transcription to obtain cDNA from the RNA template by using the Prime Script RT reagent Kit was finished. The real-time PCR assay was subsequently performed by using the SYBR® Premix Ex Taq™ II on an ABI 7500 Sequencing Detection System (Applied Biosystems, Foster City, CA). Specifically, total volume of 10 μl liquid consisted by 5 μl of SYBR® Premix Ex Taq™ II, 1 μl of diluted cDNA, 0.4 μl of mixed forward and reverse primer, and 3.6 μl of ddH2O was added into each hole of 384 PCR plate. Conditions of the 40 cycles were: 95 °C for 5 s and 60 °C for 30 s. The melting curves and reverse transcription PCR (RT-PCR) were the methods to detect the specificity of amplification product. Each target’s quantity, run in triplicate, was normalized to Actin Beta (Actb). Mouse Actb, CathepsinK (Ctsk), tartrate-resistant acid phosphatase/acid phosphatase (Trap/Acp5), nuclear factor of activated T-cells c1 (Nfatc1), AP-1 transcription factor subunit (c-Fos), Mus musculus ATPase, H + transporting, lysosomal V0 subunit D2 (Atp6v0d2), calcitonin receptor (Calcr), RUNX family transcription factor 2 (Runx2), Wnt1, Wnt10a, Wnt4, osteocalcin (Ocn), Wnt6, osteopontin (Opn), and collagen type 1 alpha 1 (Col1a1), primer sequences are listed in Table 1.

**Table 1:** Primer pairs sequences against osteoclast gene used in qPCR

Mouse gene	Forward 5′ → 3′	Reverse 5′ → 3′
*Trap/Acp5*	CAAAGAGATCGCCAGAACCG	GAGACGTTGCCAAGGTGATC
*Ctsk*	CTTCCAATACGTGCAGCAGA	TCTTCAGGGCTTTCTCGTTC
*Atp6v0d2*	GCAGAGCTGTACTTCAATGTGG	TAGTCCGTGGTCTGGAGATG
*Calcr*	TCTGCGTTCCTGAGAACACC	AAGGCGCTCTAATGGCACTT
*Nfatc1*	TGCTCCTCCTCCTGCTGCTC	GCAGAAGGTGGAGGTGCAGC
*c-Fos*	CCAGTCAAGAGCATCAGCAA	AAGTAGTGCAGCCCGGAGTA
*Runx2*	TGGCCGGGAATGATGAGAAC	GGATGAGGAATGCGCCCTAA
*Alpl*	GGGCATTGTGACTACCACTCG	CCTCTGGTGGCATCTCGTTAT
*Ocn*	GCGCTCTGTCTCTCTGACCT	TTTGTAGGCGGTCTTCAAGC
*Opn*	TTTGTAGGCGGTCTTCAAGC	GTGAGATTCGTCAGATTCATCCG
*Col1a1*	TAAGGGTCCCCAATGGTGAGA	GGGTCCCTCGACTCCTACAT
*Wnt1*	ATAGCCTCCTCCACGAACCT	GATGAACGCTGTTTCTCGGC
*Wnt4*	GAGCAACTGGCTGTACCTGG	GGAACTGGTACTGGCACTCC
*Wnt6*	GGTCACTCAAGCCTGTTCCA	CCGAAGTCCACATCGTCTCC
*Wnt10a*	CTGAACACCCGGCCATACTTC	CTGAACACCCGGCCATACTTC
*Foxc1*	CACTCGGTGCGGGAAATGT	GTGCGGTACAGAGACTGACTG
*Actb*	ACAGCAGTTGGTTGGAGCAA	ACGCGACCATCCTCCTCTTA

### Western blotting analysis

To explore the variation of the proteins on long-term activated signaling, BMMs were cultured in α-MEM containing M-CSF (30 ng/ml) and RANKL (50 ng/ml) at a density of 2 × 105 cells/well on 6-well plates. After 24 h, the BMMs were treated with or without 25 nM DZNep for 0, 1, 3, and 5 days, and total protein was obtained, respectively, on these specific time points. To determine the effect of DZNep on the protein of short-time activated phosphorylated, RAW264.7 cells were cultured in α-MEM at a density of 5 × 105 cells/well on 6-well plates. One day later, the cells were treated with the serum-free α-MEM in the presence or absence of DZNEP for 3 hand then stimulated with 50 ng/mL RANKL for 0, 10, 20, 30, and 60 min. To determine the efficiency of EZH2 shRna, BMMs were cultured in α-MEM at a density of 5 × 105 cells/well on 6-well plates. One day later, the cells were treated with negative control shRNA or different EZH2 shRNA for 2 days. To determine the efficiency of Foxc1 siRna, BMMs were cultured in α-MEM at a density of 5 × 105 cells/well on 6-well plates. One day later, the cells were treated with negative control siRNA or different Foxc1 siRNA for 2 days. To identify the role of DZNep on the EZH2 and H3K27me2 in osteoblasts, BMSC cells were cultured with α-MEM supplemented 10% FBS at a density of 5 × 105 cells/well on 6-well plates. After one day, different doses of DZNEP were treated on the cells for 4 h; then, osteoblast -cultured medium was added for another 24 h. To determine the efficiency of EZH2 shRna, BMSC were cultured in α-MEM at a density of 5 × 105 cells/well on 6-well plates. One day later, the cells were treated with negative control shRNA or different EZH2 shRNA for 2 days. To determine the efficiency of Wnt4 siRna, BMSC were cultured in α-MEM at a density of 5 × 105 cells/well on 6-well plates. One day later, the cells were treated with negative control siRNA or different Wnt4 siRNA for 2 days. Afterward, the cells were washed by the phosphate-buffered saline (PBS), and total protein of the cells was extracted by using lysate consisting of protease, phosphorylase inhibitor cocktail (Sigma-Aldrich), and radioimmunoprecipitation assay (RIPA) lysis buffer (Beyotime, Shanghai, China). After centrifuged at 13,000 × g for 15 min, the bicinchoninic acid (BCA) assay was then used to detect the concentrations of the protein in the supernatant. Finally, the supernatant proteins were diluted by 5 × SDS-sample loading buffer and separated by 4–20% SDS-PAGE before transferred to nitrocellulose filter membranes (GE Healthcare Life Sciences, Pittsburgh, PA, USA). The 5% skim milk dissolved in 1 × TBST (Tris-buffered saline with Tween 20) was used to block the membranes at room temperature for 1 h. And the primary antibodies (β-actin, 1:1000; LaminB, 1:1000; ERK, 1:1000; p-ERK, 1:1000; JNK, 1:1000; p-JNK, 1:1000; p38, 1:1000; p-p38, 1:1000; IKKβ, 1:1000; p-IKKα/β, 1:500; IκBα, 1:1000; p- IκBα, 1:1000; P65, 1:1000; p-P65, 1:500; NFATc1, 1:500; c-FOS, 1:1000; EZH2, 1:1000; H3K27me3, 1:1000; GSK-3β, 1:1000; and p-GSK-3β, 1:1000; Foxc1, 1:1000; Wnt4, 1:1000) were then incubated with the membranes together overnight at 4 °C. After TBST wash for 3 times, the secondary fluorescence antibodies were incubated without light for 1 h at room temperature and the reactivity was visualized by using Odyssey V3.0 image scanning (Li-COR. Inc., Lincoln, NE, USA). The unseparated Western blotting images are included in the supplementary material.

### Osteoblasts and osteoclasts co-cultivation

For the osteoblasts and osteoclasts co-culture system, calvarial OB isolated from day 1–3 pups were cultured for 24 h. Then the calvarial OB with complete α-MEM (α-MEM supplemented with 10% FBS, 1% penicillin/streptomycin) were seeded into 24-well plates at a density of 2 × 104 cells/well. After 3 days of cultivation, the medium was replaced by the complete α-MEM containing 50 ng/ml ascorbic acid and 5 mM β-glycerophosphate. On the same day, bone marrow monocytes were isolated from bone marrow and cultured in 10-cm dish. Another 3 days later, BMMs were seeded at a density of 4 × 104 cells/well into the 24-well plate containing the calvarial OB, and the α-MEM supplemented with 15% FBS, 1% penicillin/streptomycin, 1, 25-dihydroxy vitamin D3 (10 nM; CSN), and PGE2 (1 μM; CSN). After changed every 3 days for 2 times of the culture medium, the cells were then fixed and stained to observe the percent of the positive ALP staining area using a kit according to the manufacturer’s instructions (Beyotime, Shanghai, China). The percent of area was then quantified using ImageJ.

### Tibia cortical and trabecular bone defect mice model

This study was totally carried out in terms of the guidelines for the Ethical Conduct in the Care and Use of Nonhuman Animals in Research by the American Psychological Association. Moreover, the Animal Care and Experiment Committee of Shanghai Jiao Tong University School of Medicine approved the animal experiment of this research.

The tibia cortical and trabecular bone defect mice model was established to determine the bone healing effect of DZNep in vivo. Firstly, all mice (twelve 8-week-old C57/BL6 male mice (approximate weight 20 ± 2 g)) were anesthetized by 2% isoflurane, the right tibia was exposed, and muscles were dissociated. Then a pilot hole was first made with a burr drill point in the proximal tibia, but away from the growth plate to some extent. Then the hole was enlarged with a 1 mm reamer. Afterward, the mice were average divided into two groups: (1) DZNep group (injection with 100 μg/kg DZNep); (2) Sham group (injection with 1 × PBS). Two days after operation, 1 × PBS for sham group and DZNep for treatment group were intraperitoneally injected every two days during next two week. All mice were finally euthanized after experiment, and whole tibia bones were separated. After fixed in 4% paraformaldehyde for 24 h, the 75% ethanol was then used to soak the specimens for further histological and radiographic analysis.

### Micro-computed tomography scanning

High-resolution micro-CT (μCT-100, SCANCO Medical AG, Switzerland) was used to perform the micro-computed tomography (CT) scanning. Scans were conducted in 75% ethanol and used an X-ray intensity of 200 uA, an X-ray tube potential of 70 kVp, and an integration time of 300 ms. For analysis bone mass of defect section, a region of defect section trabecular bone was contoured, starting from the first defect section level away from proximal end of the distal femoral growth plate. Femoral trabecular bone was thresholded at 211 per mille. The indicators of bone microstructure (bone volume/tissue volume (BV/TV), trabecular thickness (Tb.Th), trabecula number (Tb.N), trabecula separation (Tb.Sp), and bone surface/ bone volume (BS/BV)) were measured by software (Version: 6.5–3, SCANCO Medical AG, Switzerland) through evaluating and analyzing the three-dimensional region of interest (ROI).

### Histological analysis

After fixing in 4% paraformaldehyde for 48 h, the 10% EDTA was used to decalcify the fixed tibias for 21 days. Then the tissues were processed by dehydrating in different concentrations of ethanol, infiltrating with xylene, and embedding with paraffin. Hematoxylin and eosin (H&E) and TRAP staining were then performed on these prepared histological sections. As for immunofluorescent staining, slices were processed in a sequence of deparaffinization, hydration, antigen retrieval, permeabilization, blocking, primary antibody incubation (OCN, USA, Affinity; dilution 1:100), secondary antibody incubation, and nuclear staining finally. Immunohistochemical (IHC) staining was accomplished with antibodies against OCN (USA, Affinity; dilution 1:100) [29]. The specimens were observed and photographed under the high-quality microscope (Leica DM4000B). Each sample’s number of osteoclasts and TRAP-positive multinucleated osteoclasts per field (Oc.S/BS) were calculated.

### Statistical analysis

The results were analyzed by using Prism 8 (GraphPad Software Inc, San Diego, CA, USA). The data were uniformly presented in the form of median and interquartile range. The comparisons between experimental and control groups were made by the 2-tailed, unpaired Student’s t test, one-way ANOVA with Tukey’s post hoc test. Statistical significance was determined to be at *p < 0.05; **p < 0.01; ***p < 0.001; ****p < 0.0001.

## Results

### DZNep promoted RANKL-induced osteoclastogenesis in vitro

**Fig. 1:**
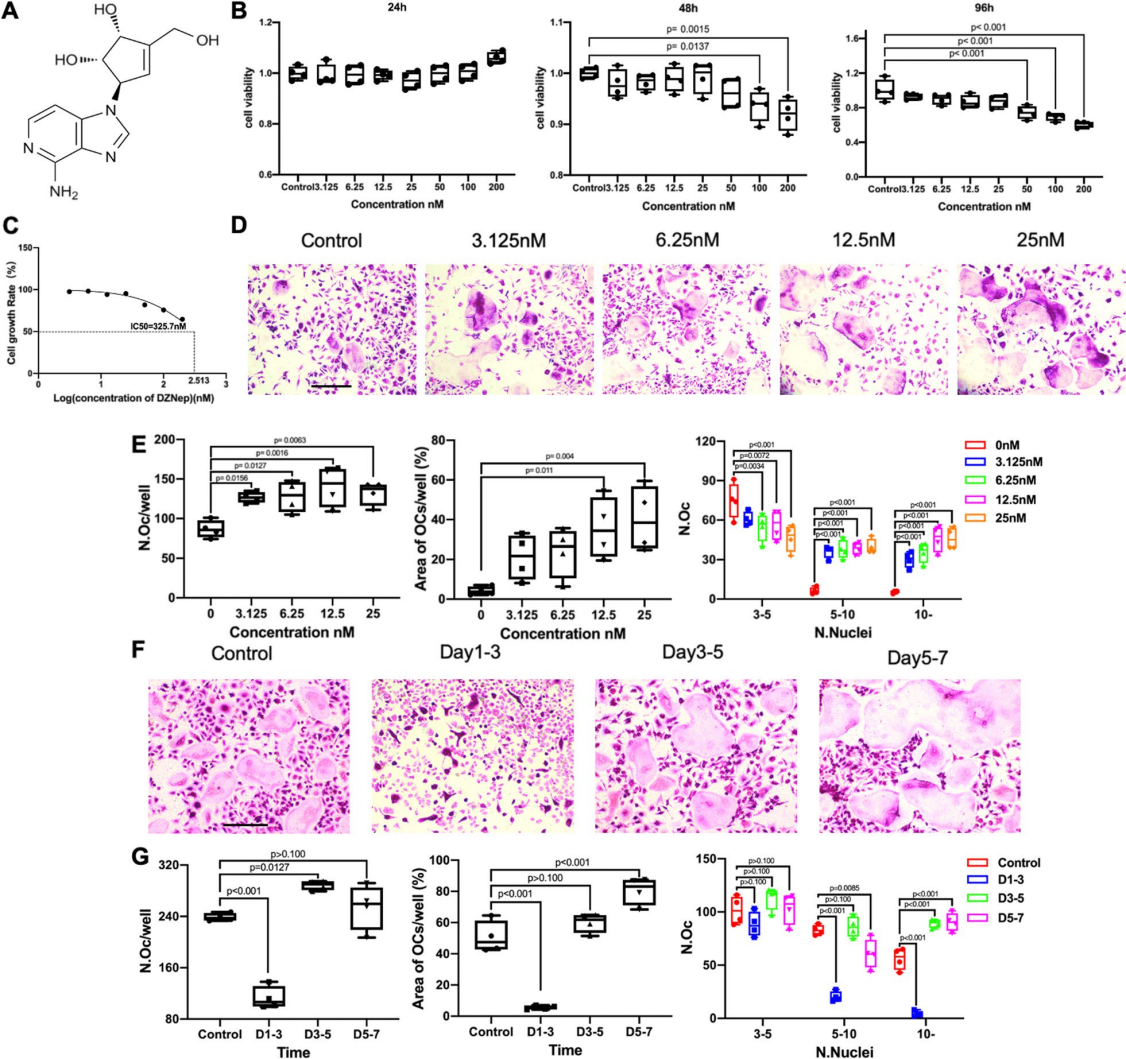
DZNep promoted RANKL-induced osteoclastogenesis without cytotoxic effects in vitro. **A** The structure of DZNep. **B** Cell viability of DZNep-treated BMMs was tested by CCK-8 at 24, 48, and 96 h. **C** IC50 value obtained for the activity of DZNep against BMMs. **D** BMMs were treated with different concentrations of DZNep and stimulated by M-CSF (30 ng/ml) and RANKL (50 ng/ml) for 5 days. Then the cells were fixed with 4% paraformaldehyde and stained for TRAP. Scale bar = 100 μm. **E** Quantification of TRAP-positive multinuclear cells, number of osteoclasts, area of osteoclasts, and osteoclast size (*p < 0.05; **p < 0.01; ***p < 0.001; ****p < 0.0001). **F** TRAP-positive BMMs following the treatment with 25 nM DZNep for indicated days during osteoclastogenesis. Scale bar = 100 μm. **G** Quantification of TRAP-positive multinuclear cells, number of osteoclasts, area of osteoclasts, and osteoclast size (*p < 0.05; **p < 0.01; ***p < 0.001; ****p < 0.0001). Data are expressed as median and interquartile range, *n* = 4–6

The cytotoxicity effect of DZNep (Fig. 1A) on primary BMMs was investigated. As shown in Fig. 1B, no cytotoxicity was found at the concentration of 25 nM. The inhibitory concentration (IC50) value of DZNep was examined to be 325.7 nM at 96 h (Fig. 1C). To further explore the role of DZNep on osteoclastogenesis in vitro, BMMs were cultured with RANKL, M-CSF and DZNep at concentrations less than or equal to 25 nM. A little number of multinucleated trap-positive osteoclasts were observed in the control group after 5-day stimulation. However, the experimental groups increased the number of mature osteoclasts in a DZNep dose-dependent manner. The number of mature osteoclast nuclei also increased in a dose-dependent manner significantly when compared with the control group (Fig. 1D, E). Furthermore, we added 25 nM DZNep into the osteoclast-inductive medium at specific time points. Comparing with the control group, DZNep increased the number of osteoclasts at the middle stage (day 3–5). More osteoclasts with more than 10 nuclei could be found at the middle stage and final stage of osteoclast differentiation (day 3–5, day 5–7) compared with control (Fig. 1F, G). Therefore, DZNep enhanced osteoclastogenesis in vitro.

### DZNep increased osteoclast resorption capacity in vitro

**Fig. 2:**
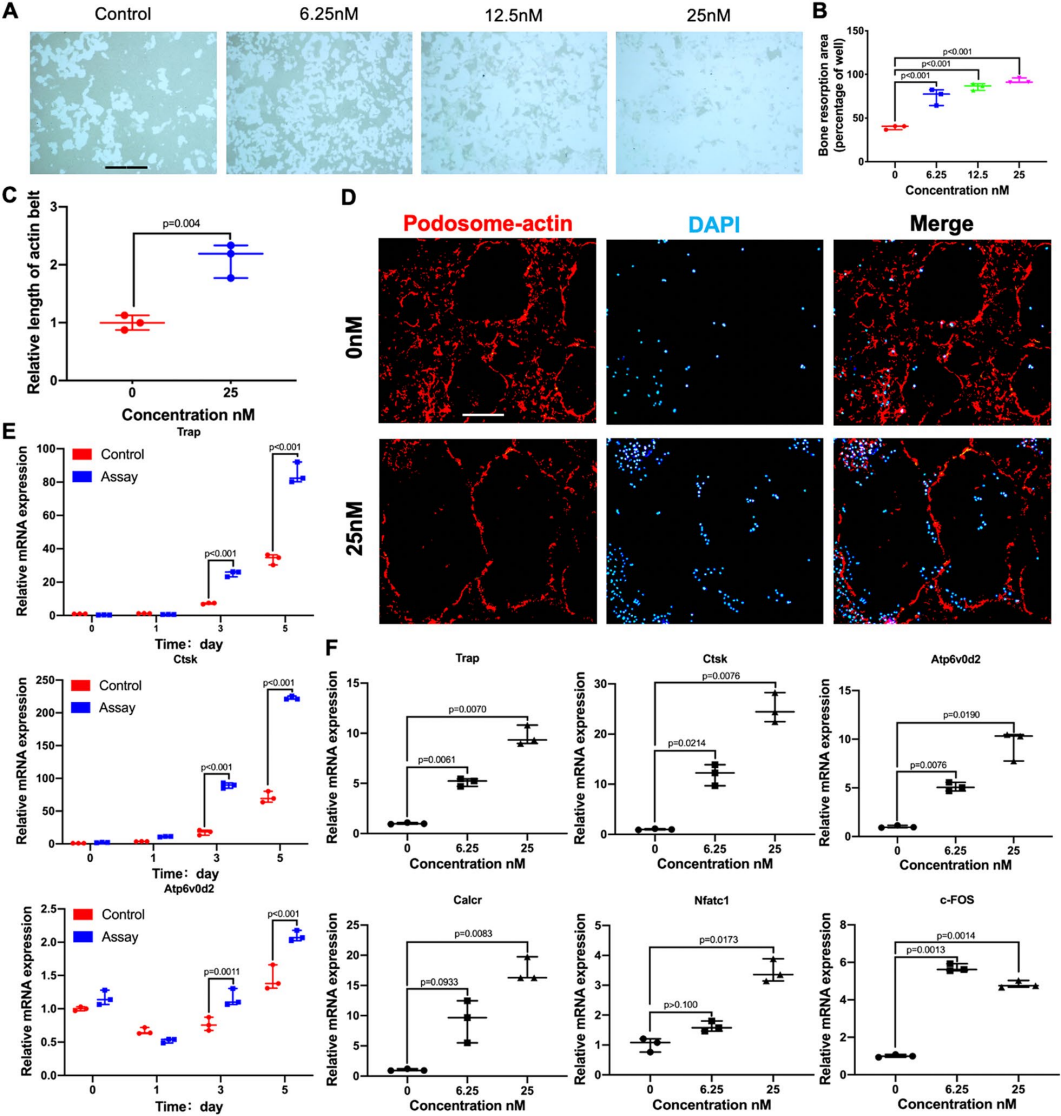
DZNep promoted osteoclastic bone resorption, osteoclast precursor cell fusion, and the expression of osteoclast-specific genes in vitro. **A** BMMs were seeded onto the hydroxyapatite-coated Osteo Assay plates and cultured with M-CSF (30 ng/mL), RANKL (50 ng/mL), and with 0, 6.25, 12.5, and 25 nM DZNep for 5 days. Scale bar = 200 μm. **B** Quantification of bone resorption area was performed by ImageJ (*p < 0.05; **p < 0.01; ***p < 0.001; ****p < 0.0001). **C** The perimeter of the actin ring was measured using ImageJ. **D** Representative immunofluorescence images for podosome actin belt formation. Scale bar = 20 μm.(*p < 0.05; **p < 0.01; ***p < 0.001). **E** Expression of the osteoclast-specific genes Trap, Ctsk, and Atp6v0d2 in BMMs treated with 25 nM DZNep, M-CSF (30 ng/mL), and RANKL (50 ng/mL) for 0, 1, 3, and 5 days. **F** Expression of the osteoclast-specific genes Trap, Ctsk, Atp6v0d2, Calcr, Nfatc1, and c-Fos in BMMs treated with M-CSF (30 ng/mL), RANKL (50 ng/mL), and indicated DZNep concentrations for 5 days. Gene expression was analyzed by real-time PCR. mRNA expression levels were normalized relative to the expression of β-actin (*p < 0.05; **p < 0.01; ***p < 0.001; ****p < 0.0001). Data are expressed as median and interquartile range, *n* = 3

To determine the function of DZNep on osteoclastic bone resorption, different concentrations of DZNep were added after primary BMMs were plated on the hydroxyapatite-coated Osteo Assay plates (Fig. 2A). Bone resorption pits were markedly induced. When treated with DZNEP at 25 nM, more than 30% bone resorption area was observed compared with the control group (Fig. 2B).

### DZNep induced osteoclast podosome belt formation in vitro

Cytoskeletal podosome belt formation is an indispensable part of osteoclastic bone resorption [30]. Here we examined the role of DZNep on the formation of osteoclast cytoskeletal podosome belt. DZNep promoted the podosome actin belt formation of osteoclast at the concentration of 25 nM compared with the control group (Fig. 2C, D). To sum up, these data suggested that DZNep substantially increased osteoclastic bone resorption capacity along with osteoclast podosome belt formation in vitro.

### DZNep upregulated the osteoclast-related genes expression in vitro

In order to better comprehend the effect of DZNep on osteoclasts, we analyzed the expression of several vital osteoclast-related genes by real-time PCR (qPCR). The genes markedly upregulated during the process of osteoclast formation were measured, such as Trap, Ctsk, Atp6v0d2. After DZNep was added at the dose of 25 nM, these genes expression was significantly enhanced in a time-dependent manner (Fig. 2E). Meanwhile, the expression of Calcr, c-FOS, and Nfatc1 along with genes mentioned above was upregulated in a dose-dependent manner after DZNep was added (Fig. 2F). Together, these data further confirmed that DZNEP promotes osteoclastogenesis in vitro.

### DZNep promoted osteoclastogenesis by activating the NF-kB signaling pathway

**Fig. 3:**
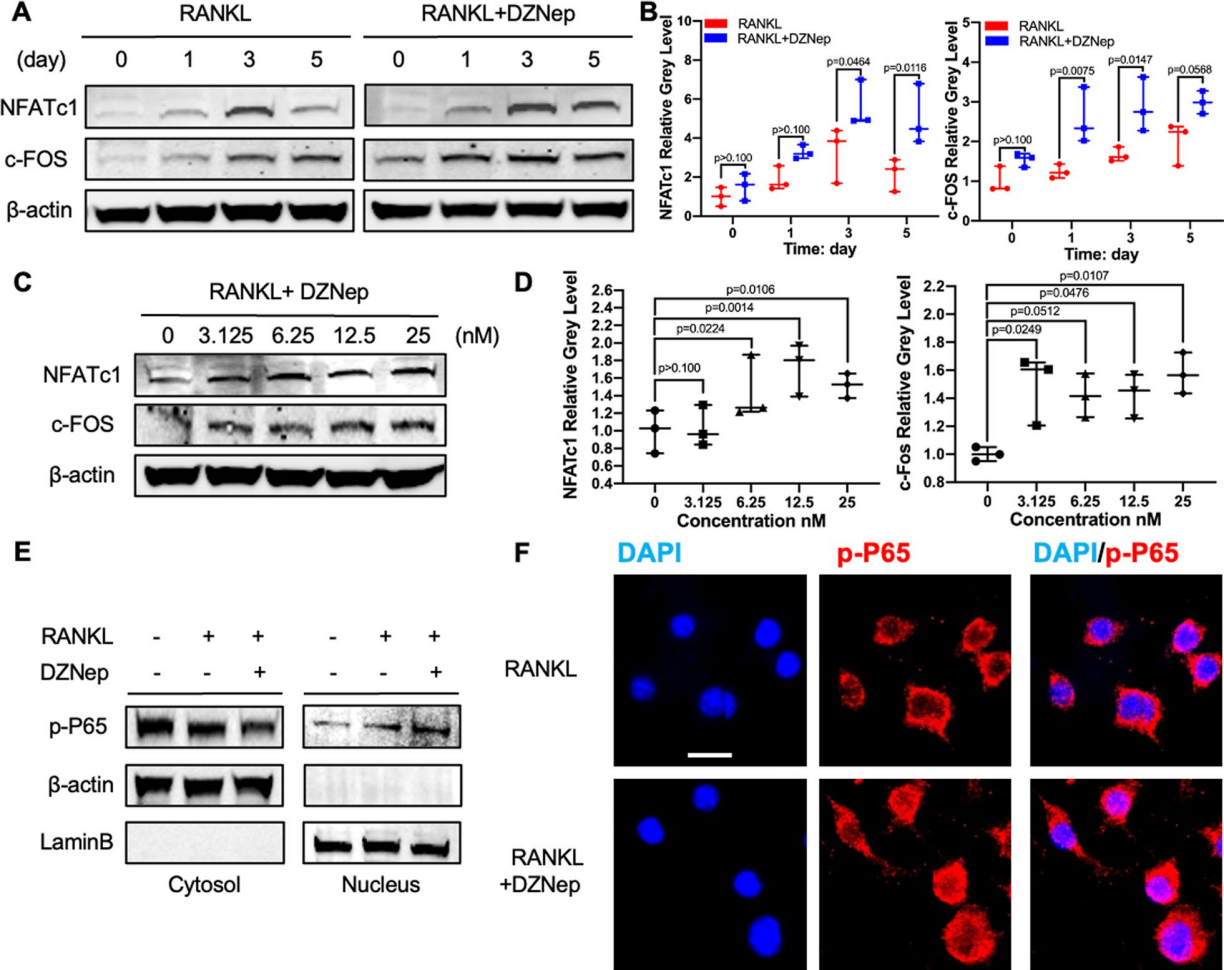
DZNep promoted osteoclast formation by inducing the p-P65 nuclear translocation. **A** NFATc1 and c-Fos expression levels in BMMs treated with or without 25 nM of DZNep for 0, 1, 3, and 5 days. **B** The gray levels of NFATc1 and c-Fos were quantified and normalized to β-actin using ImageJ. **C** NFATc1 and c-Fos expression levels in BMMs stimulated with M-CSF (30 ng/mL), and RANKL (50 ng/mL) for 5 days in presence of a various concentration of DZNep. **D** The gray levels of NFATc1 and c-Fos were quantified and normalized to β-actin using ImageJ. **E** BMMs were pretreated with 25 nM of DZNep for 3 h before the addition of RANKL for 30 min. Nuclear translocation of p-P65 was analyzed using western blotting. **F** RAW264.7 cells were pretreated with 25 nM DZNep for 2 h, and then RANKL was added for 1 h. Nuclear translocation of p-P65 was then visualized by immunofluorescence. Scale bar = 20 nM (*p < 0.05; **p < 0.01; ***p < 0.001; ****p < 0.0001). Data are expressed as median and interquartile range, *n* = 3

**Fig. 4:**
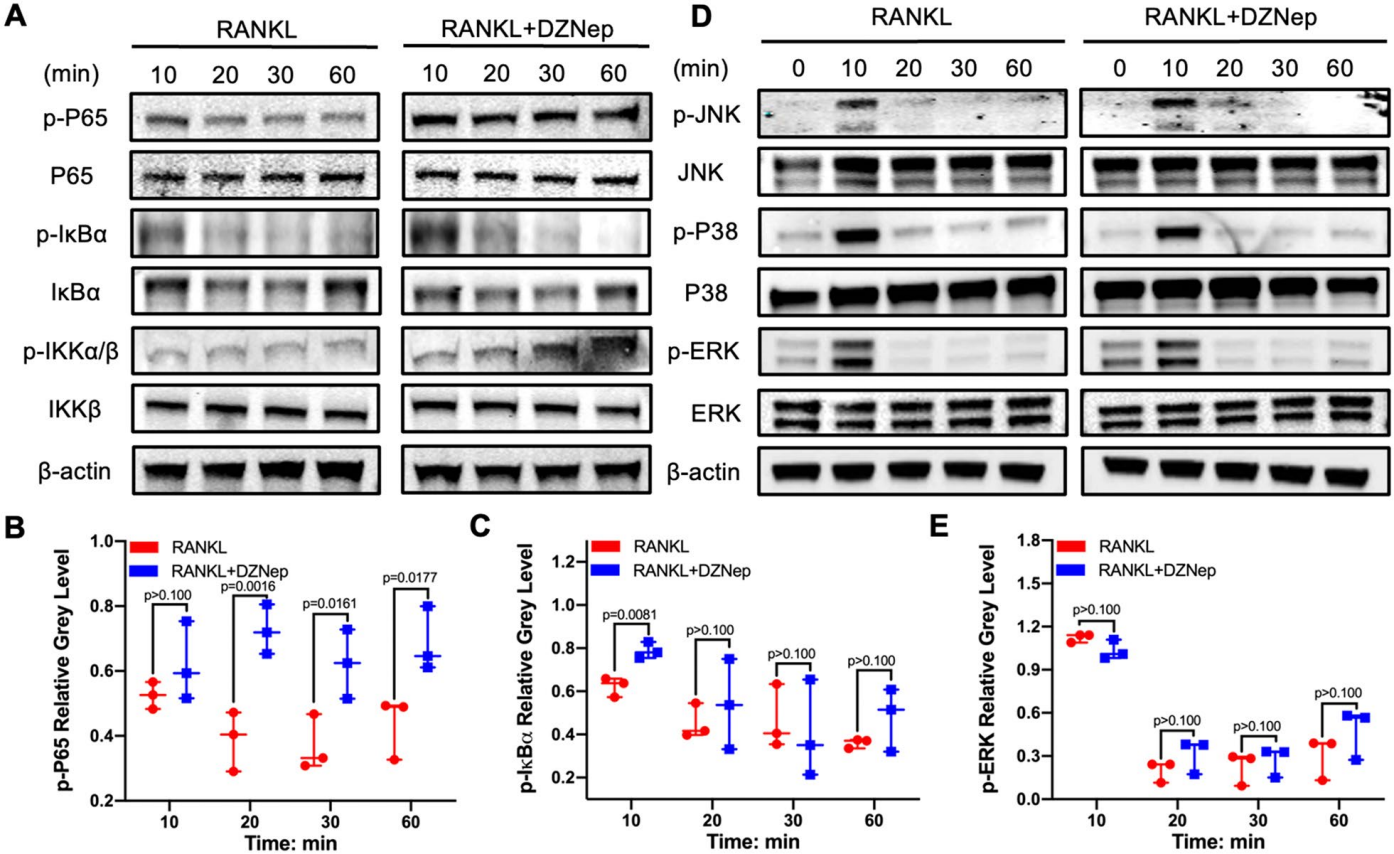
DZNep promoted the p-P65 nuclear by facilitating the phosphorylation of IKKα/β, and the degradation of IκBα instead of the MAPK pathway. **A** RAW264.7 cells were pretreated with 25 nM DZNep for 2 h before the stimulation with RANKL for specific times, and the levels of NF-κB signaling pathway proteins were determined. **B** The gray levels of p-P65 were quantified and normalized to β-actin using ImageJ. **C** The gray levels of p-IκB***a*** were quantified and normalized to β-actin using ImageJ. **D** RAW264.7 cells were pretreated with 25 nM DZNep for 2 h before the RANKL was added on different time points, and the expression of MAPK signaling pathway proteins was explored. **E** The gray levels of p-ERK were quantified and normalized to β-actin using ImageJ (*p < 0.05; **p < 0.01; ***p < 0.001; ****p < 0.0001). Data are expressed as median and interquartile range, *n* = 3

To clarify the detailed mechanisms about the DZNep-induced promotion of osteoclast formation, we first investigated the effect of DZNep on the c-FOS and NFATc1 which is the downstream of c-FOS and has been proved to be one of the most significant transcription factors for osteoclast differentiation. BMMs were cultured in medium added with RANKL, with or without DZNep for 0, 1, 3, and 5 days. The expression of c-FOS and NFATc1 was both gradually increased over time in the control group and was further enhanced after DZNep was added at the concentration of 25 nM (Fig. 3A, B). Also, the c-FOS and NFATc1 expression was upregulated after BMMs were treated with RANKL, with DZNep in a concentration-dependent manner for 5 days (Fig. 3C, D). We further examined whether the enhanced c-FOS and NFATc1 expression in osteoclast was also through the activated NF-κB pathway. Consistent with our hypothesis, our western blotting analyses and immunofluorescence staining of cytoplasm and nuclear proteins showed the enhancement of NF-κB nuclear translocation upon DZNep treatment (Fig. 3E, F). Previous literature demonstrated that DZNep could upregulate the NF-κB nuclear translocation by promoting the IκB phosphorylation in mice airway smooth muscle cells and human basal-like breast cancer cells [12, 13]. After that, we examined the transduction factors of classical NF-κB signaling pathways, which mainly include the inhibitor of nuclear factor kappa-B kinase (IKK), and inhibitor of nuclear factor kappa-B (IκB), and NF-κB. We observed the phosphorylation of IKK, IκB, and p65 was further promoted over time with DZNep treatment (Fig. 4A, B, and C). We also examined the influence of DZNep on another important signaling pathways, the MAPKs signaling pathways, for osteoclast formation. Our data show that the phosphorylation of JNK, p38, and ERK was comparable in both the control and the DZNep-treated groups (Fig. 4D, E). Collectively, these results indicated that DZNep promoted the phosphorylation of IKK, and IκB, accelerated the degradation of IκB, and further stimulated the p65 nuclear translocation during osteoclast formation.

### DZNep activated the NF-kB signaling pathway through the EZH2-H3K27me3-Foxc1 axis

**Fig. 5:**
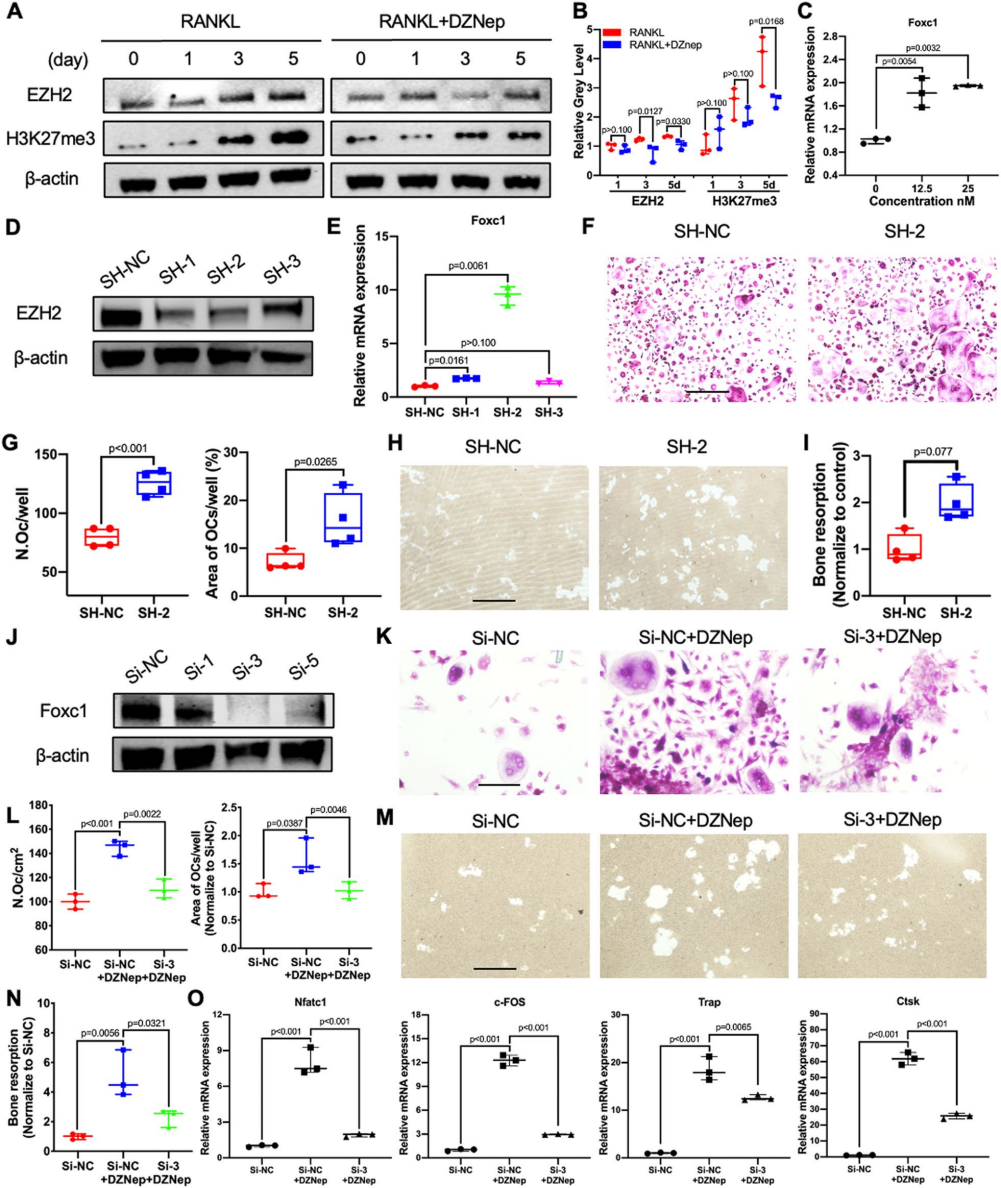
DZNep activated the NF-kB signaling pathway through EZH2-H3K27me3-Foxc1 axis. **A** BMMs cells were treated with or without 25 nM DZNep for 0, 1, 3, 5 days accompanied the stimulation of RANKL, and the levels of EZH2 and H3K27me3 were determined. **B** The gray levels of EZH2 and H3K27me3 were quantified and normalized to β-actin using ImageJ. **C** Expression of the Foxc1 in BMMs treated with M-CSF (30 ng/mL), RANKL (50 ng/mL), and indicated DZNep concentrations for 5 days. **D** BMMs cells were treated with negative control or different shRna of EZH2, and the levels of EZH2 were determined. **E** Expression of the Foxc1 in BMMs after treated with negative control or different shRna of EZH2. **F** BMMs were treated with negative control or shRna-2 of EZH2 and stimulated by M-CSF (30 ng/ml) and RANKL (50 ng/ml) for 5 days. Then the cells were fixed with 4% paraformaldehyde and stained for TRAP. Scale bar = 20 μm. **G** Quantification of number of osteoclasts, area of osteoclasts. **H** BMMs were seeded onto the hydroxyapatite-coated Osteo Assay plates and cultured with M-CSF (30 ng/mL), RANKL (50 ng/mL), and with negative control or shRna-2 of EZH2 for 7 days. Scale bar = 20 μm. **I** Quantification of bone resorption area was performed by ImageJ. **J** BMMs cells were treated with negative control or different siRna of Foxc1, and the levels of Foxc1 were determined. **K** BMMs were treated with siRna-NC, siRna-NC with DZNep, or siRna-3 of Foxc1 with DZNep and stimulated by M-CSF (30 ng/ml) and RANKL (50 ng/ml) for 5 days. Then the cells were fixed with 4% paraformaldehyde and stained for TRAP. Scale bar = 5 μm. **L** Quantification of number of osteoclasts, area of osteoclasts. **M** BMMs were seeded onto the hydroxyapatite-coated Osteo Assay plates and cultured with M-CSF (30 ng/mL), RANKL (50 ng/mL), and with siRna-NC, siRna-NC with DZNep or siRna-3 of Foxc1 with DZNep for 7 days. Scale bar = 10 μm. **N** Quantification of bone resorption area was performed by ImageJ. **O** Expression of the osteoclast-specific genes Nfatc1, c-Fos, Trap, and Ctsk in BMMs treated with siRna-NC, siRna-NC with DZNep, or siRna-3 of Foxc1 with DZNep and stimulated by M-CSF (30 ng/ml) and RANKL (50 ng/ml) for 5 days (*p < 0.05; **p < 0.01; ***p < 0.001; ****p < 0.0001). Data are expressed as median and interquartile range, *n* = 3–4

As previously introduced that the inhibition of EZH2 and subsequently downregulated H3K27me3 resulted in the transcriptional upregulation of Foxc1, which activates the phosphorylation of the IκB [11–13]. We examined the role of DZNep on the EZH2-H3K27me3-Foxc1 axis. BMMs were cultured in medium with RANKL, with or without DZNep for 0, 1, 3, and 5 days. The EZH2 and H3K27me3 expression was decreased at a time manner after DZNep was added at the concentration of 25 nM when comparing with the control group (Fig. 5A, B). Also, the upregulated mRNA expression of Foxc1 was observed in a concentration-dependent manner after BMMs RANKL, and DZNep treatment for 5 days (Fig. 5C). As predicted, our further EZH2 silencing experiment results were in accordance with that inhibition of EZH2 induces the expression of Foxc 1, which could be most obviously observed in the group treated with shRna-2 of EZH2 (Fig. 5D, E). Moreover, the experimental groups added with shRNA also increased the area and number of mature osteoclasts when compared with the negative shRNA-treated group (Fig. 5F, G). Matching with it, the bone resorption capacity was enhanced in the shRNA-treated group (Fig. 5H, I). Furthermore, the group treated with Foxc1 siRna-3, the most efficient one (Fig. 5J), and DZNep partly reversed the area and number of mature osteoclasts when compared with the negative siRNA- and DZNep-treated group (Fig. 5K, L). Matching with it, the bone resorption capacity and the osteoclast-specific genes expression were also reduced in the siRNA-3 and DZNep-treated group when compared with the negative siRNA and DZNep-treated group (Fig. 5M-O). These results illustrated that DZNep activated the NF-kB signaling pathway partly through the EZH2-H3K27me3-Foxc1 axis, which mediating the enhanced osteoclastogenesis.

### DZNep induced osteogenesis in vitro

**Fig. 6:**
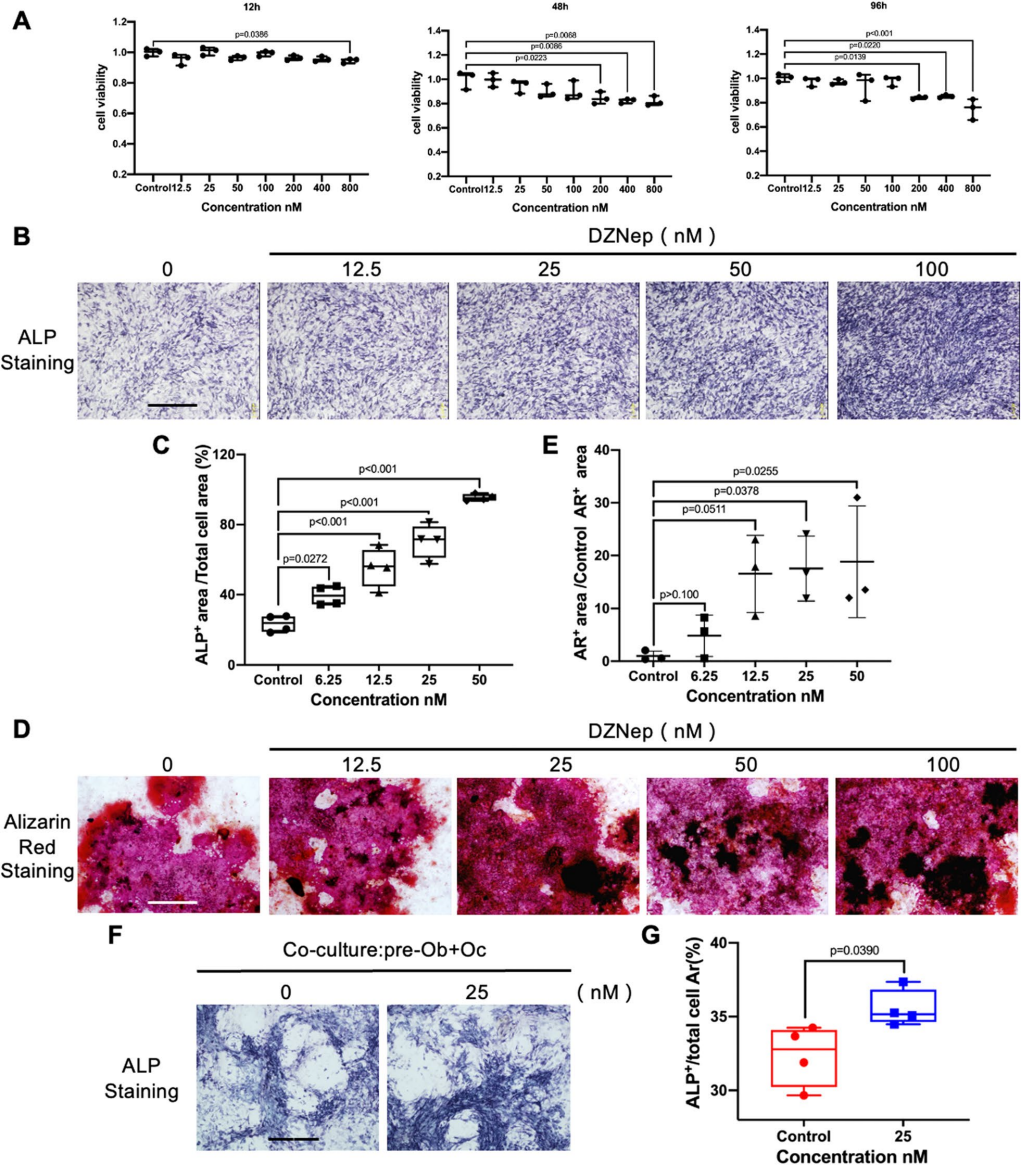
The effects of DZNep on osteoblastogenesis and co-cultivation system. **A** Cell viability of DZNep-treated BMSC was tested by CCK-8 at 12, 48, and 96 h. **B** BMSC were treated with osteoblast-cultured medium and with different concentrations of DZNep for 7 days. Then the ALP staining was performed after cells were fixed with 4% paraformaldehyde. Scale bar = 20 μm. **C** Percentage of ALP + area of total area was quantified by ImageJ (*p < 0.05; **p < 0.01; ***p < 0.001; ****p < 0.0001). **D** The Alizarin Red staining was performed after BMSC were treated with osteoblast-cultured medium and with indicated concentrations of DZNep for 28 days. Scale bar = 50 μm. **E** Quantification of area of calcium deposition in the extracellular matrix (*p < 0.05; **p < 0.01; ***p < 0.001; ****p < 0.0001). **F** ALP was detected after BMMs and osteoblast precursor cells (calvarial OB-derived) were co-cultivated in presence or absence of DZNep for 7 days. **G** Percentage of ALP + area of total area was analyzed by ImageJ (*p < 0.05; **p < 0.01; ***p < 0.001; ****p < 0.0001). Data are expressed as median and interquartile range, *n* = 3–4

We further investigated the effect of DZNep on osteogenesis. We first performed the cytotoxicity experiment of DZNep on primary BMSC at 12, 48, and 96 h. Results showed that no cytotoxicity was found at the concentration of 100 nM (Fig. 6A). After that, osteoblasts-cultured medium was added on primary BMSC for 7 days and 28 days, with different concentrations of DZNep. ALP staining showed that osteoblast differentiation was promoted by DZNep, especially on the concentration of 50 nM (Fig. 6B, C). Further Alizarin red staining also demonstrated that osteoblast mineralization was enhanced in a dose-dependent manner (Fig. 6D, E). Besides, co-culture system which contained both osteoclasts and pre-osteoblasts also revealed that DZNep still preserves the effect of promoting osteogenesis (Fig. 6F, G). Taken together, DZNep promoted osteogenesis in vitro.

### DZNep promoted osteogenesis through EZH2-H3K27me3-Wnt4 axis in vitro

**Fig. 7:**
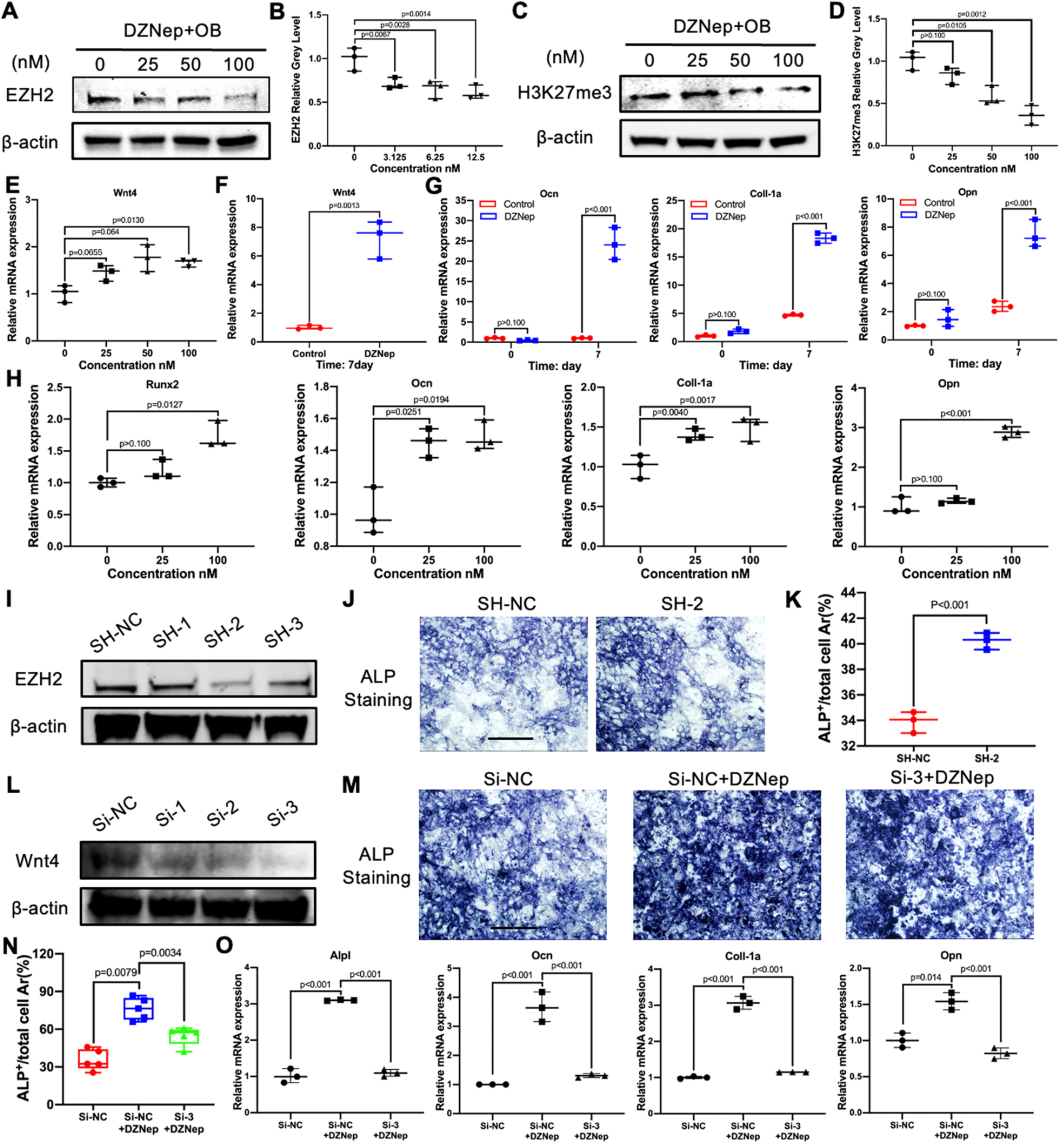
DZNep promoted osteoblasts formation through the EZH2-H3K27me3-Wnt4 axis. **A** EZH2 expression levels in BMSC treated with 0, 25, 50, 100 nM of DZNep combined with osteoblast-cultured medium for 24 h. **B** The gray levels of EZH2 were quantified and normalized to β-actin using ImageJ. **C** H3K27me3 expression levels in BMSC treated with 0, 25, 50, 100 nM of DZNep combined with osteoblast-cultured medium for 24 h. **D** The gray levels of H3K27me3 were quantified and normalized to β-actin using ImageJ. **E** Expression of the wnt signaling pathway-specific genes Wnt4 in BMSC treated with 0, 25, 50, 100 nM of DZNep combined with osteoblast-cultured medium for 24 h. **F** Expression of the wnt signaling pathway-specific genes Wnt4 in BMSC treated with or without 100 nM of DZNep combined with osteoblast-cultured medium for 7 days. **G** Expression of the osteoblast-genes Runx2, Ocn, Coll-1a, and Opn in BMSC treated with or without 100 nM of DZNep combined with osteoblast culture medium for 0 and 7 days. **H** Expression of the osteoblast-genes Runx2, Ocn, Coll-1a, and Opn in BMSC treated with 0, 25, 50, 100 nM of DZNep combined with osteoblast-cultured medium for 24 h. **I** BMSCs cells were treated with negative control or different shRna of EZH2, and the levels of EZH2 were determined. **J** BMSCs were treated with negative control or shRna-2 of EZH2 and stimulated by osteoblast-cultured medium for 7 days. Then the ALP staining was performed after cells were fixed with 4% paraformaldehyde. Scale bar = 20 μm. **K** Percentage of ALP^+^ area of total area was quantified by ImageJ. **L** BMSCs cells were treated with negative control or different siRna of Wnt4, and the levels of Wnt4 were determined. **M** BMSCs were treated with siRna-NC, siRna-NC with DZNep or siRna-3 of Wnt4 with DZNep and stimulated by osteoblast-cultured medium for 7 days. Then the ALP staining was performed after cells were fixed with 4% paraformaldehyde. Scale bar = 20 μm. **N** Percentage of ALP^+^ area of total area was quantified by ImageJ. **O** Expression of the osteoblast-specific genes Alpl, Ocn, Coll-1a, Opn in BMSCs treated with siRna-NC, siRna-NC with DZNep or siRna-3 of Wnt4 with DZNep and stimulated by osteoblast-cultured medium for 7 days. Gene expression was analyzed by real-time PCR. mRNA expression levels were normalized relative to the expression of β-actin (*p < 0.05; **p < 0.01; ***p < 0.001; ****p < 0.0001). Data are expressed as median and interquartile range, *n* = 3–5

To excavate unknown mechanisms on the enhanced osteogenesis of DZNep, we firstly performed the western blotting experiment on the proteins extracted from the primary BMSC stimulated with different concentrations of DZNep for 24 h. Results showed that the EZH2 and H3k27me3 expression was dose-dependent decreased, compared with the control group (Fig. 7A–D). After that, important Wnt signaling pathway-related genes in a short-time stimulation were examined by qPCR. Interestingly, Wnt4 as well as Wnt1, Wnt6a, and Wnt10, which had been reported upregulated via the DZNep-EZH2-H3K27me3 axis, was increased in a concentration-dependent manner (Fig. 7E, Additional file 1: Figure S1A). Moreover, the expression of these genes after the osteoblast-cultured medium added for 7 days was obviously upregulated as well (Fig. 7F, Additional file 1: Figure S1B), while the canonical upstream activator, the GSK-3β, of β-catenin had no significant change on the level of phosphorylation (Additional file 1: Figure S2A, B). However, the downstream osteoblast-specified genes Runx2, Ocn, Opn, Col1a1 were enhanced in a time-dependent and dose-dependent manner (Fig. 7G, H). The positive regulation of osteogenesis by DZNep-targeted EZH2 was further proved by the EZH2 silencing experiment, which could be most obviously observed in the group treated with shRna-2 of EZH2 (Fig. 7I). The experimental groups added with shRNA also increased the percent of ALP staining area when compared with the negative shRNA-treated group (Fig. 7J, K). More convincingly, the group treated with siRna-3, the most efficient one (Fig. 7L), and DZNep partly reversed the percent of ALP staining area when compared with the negative siRNA and DZNep-treated group (Fig. 7M, N). Matching with it, the osteoblast-specific genes expression was also reduced in the siRNA-3- and DZNep-treated group when compared with the negative siRNA- and DZNep-treated group (Fig. 7O). To sum up, DZNep promoted osteogenesis partly through the EZH2-H3K27me3-Wnt4 axis in vitro.

### Administration of DZNep facilitated the repair of bone defect in vivo

**Fig. 8:**
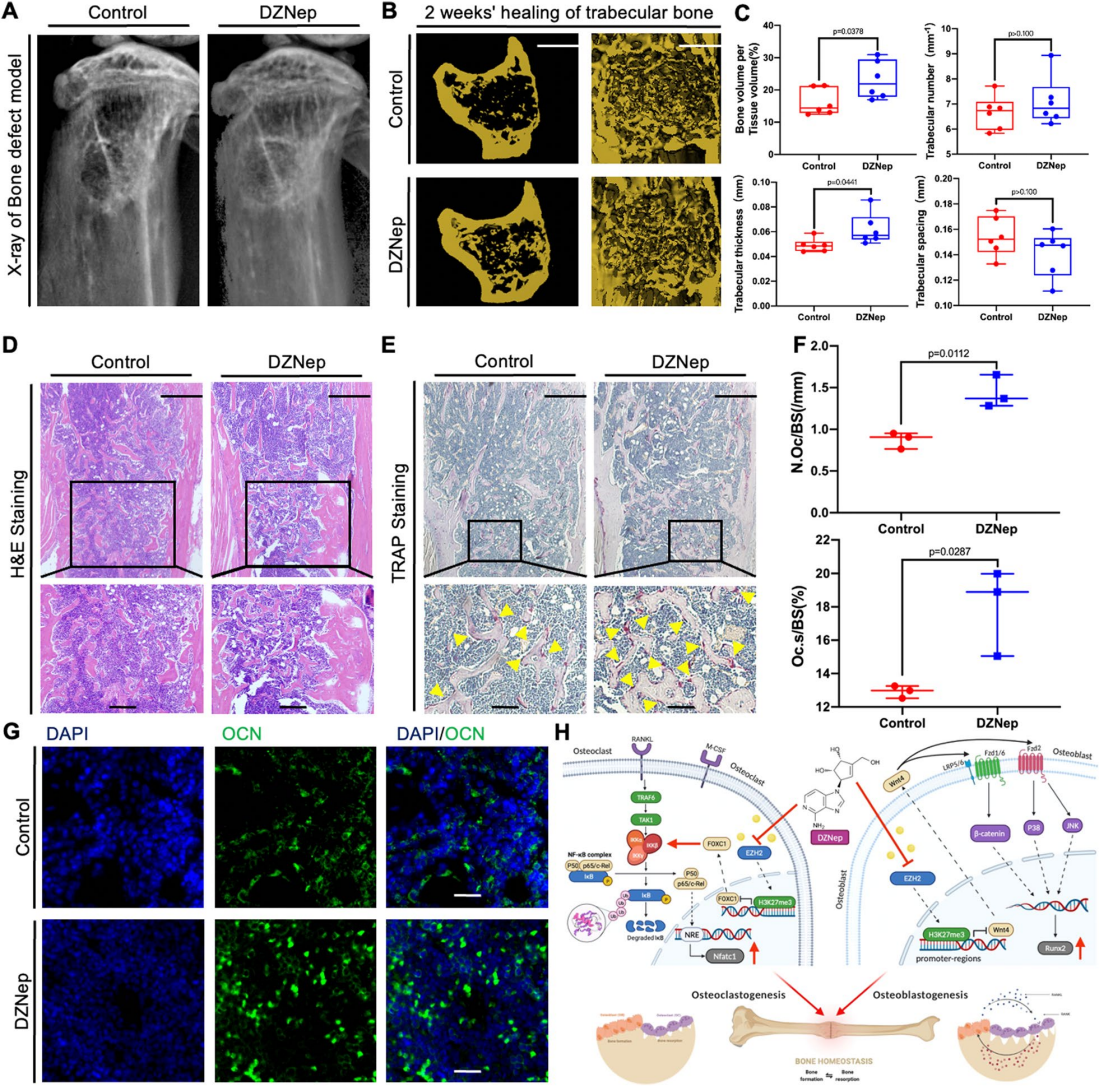
DZNep showed the therapeutic effect on bone defect in vivo. **A** The defect tibias of mice treated with or without DZNep were scanned with animal X-ray, and two-dimensional images are presented. **B** The fixed tibias of bone defect mice divided into two groups and treated for 2 weeks were analyzed by mCT, and three-dimensional reconstructed images are presented. Scale bar = 50 μm (*p < 0.05; **p < 0.01; ***p < 0.001; ****p < 0.0001). **C** Microstructural indices include bone volume/tissue volume (BV/TV), trabecular number (Tb.N), trabecular thickness(Tb.Th), and trabecular separation (Tb.Sp) obtained by analyzing the two mouse groups are presented. **D** H&E (magnification × 4 and × 10) staining, respectively, of the defect tibia sections. Scale bar = 25 μm and 10 μm. **E** TRAP (magnification × 4 and × 10) staining, respectively, of the defect tibia sections. Scale bar = 25 μm and 5 μm. **F** The number of TRAP-positive cells and OcS/BS%. **G** Immunofluorescence staining, respectively, of Ocn in the defect tibiae sections. Scale bar = 25 μm. **H** Schematic representation of the molecular mechanism of the article (created with BioRender.com). Data are expressed as median and interquartile range, *n* = 3–6

Given the enhanced osteogenesis and osteoclastogenesis effect in vitro, we further examined whether DZNep can facilitate bone defect repair in vivo. We first examined the animal X-ray in mice treated with or without DZNep for two weeks after bone defect. Image results showed that there were more bone mineral density in the treated group when compared with the control group (Fig. 8A). Consistent with the two-dimensional (2D) images, the three-dimensional (3D) reconstruction of the micro-CT scanning also indicated the accelerated repair in mice with bone defect (Fig. 8B). Further quantitative analysis demonstrated that the BV/TV and the trabecular thickness (Tb.th) were prominently increased in the treated group (Fig. 8C). Moreover, the H&E-stained sections obtained from the treated group indicated more significant bone repair in comparison with the control group (Fig. 8D). In vivo TRAP staining shows the same results as in vitro that the administrated group has more number of TRAP-positive multinucleated osteoclasts compared with the vehicle-treated group (Fig. 8E, F). Meanwhile, the osteocalcin (OCN) expression, which mainly represents the formation of matured osteoblasts, was also increased in the DZNep-treated group (Fig. 8G, Additional file 1: Figure S3). These results illustrated that administration of DZNep facilitated the repair of bone defect In vivo.

## Discussion

DZNep was reported as a potential compound to treat many kinds of tumors. Here in our study, we demonstrated that DZNep is also effective in enhancing bone defect repair, which partly credit to the enhanced osteoblast differentiation and osteoclast formation. Caner bone metastasis is commonly accompanied by destruction of bone [31]. The protective effect of DZNep may further protect the bone during the treatment of tumors. Therefore, this finding may further promote the use of DZNep for bone metastasis tumors.

The balance between osteoclastic bone resorption and osteoblastic bone formation maintains the homeostasis of bone. The differentiation and mineralization of osteoblasts had been proved to be essential for fracture and bone defect healing [32]. Meanwhile, osteoclast formation facilitated late-stage callus remodeling [33]. During the past decades, many methods, like drugs, compounds, factors, and biomaterials, have been made in the treatment of bone fractures and defects [34–36]. Therefore, compounds that can enhance osteogenesis and osteoclastogenesis are required for the treatment of the bone fracture and defect. In our present research, we demonstrated that DZNep could strongly promote RANKL-induced osteoclastogenesis. Meanwhile, the formation of osteoclast podosome belt and bone resorption was also enhanced. The underlying mechanisms were also revealed. We primarily investigated the nuclear translocation of p-p65, which binds to the Nfatc1 promoter and induces osteoclast formation [37, 38]. Our data showed that the nuclear translocation of p-p65 was specifically activated after treated with DZNep. The canonical activation of nuclear translocation and NF-kB p65 subunits phosphorylation began from the binding between RANKL and the receptor RANK [39]. Then the phosphorylation of TAK1 and downstream molecules IKKβ, IκB was activated successively, which finally resulted in the IκB proteasomal degradation and p-p65 nuclear translocation [40]. And in the study of DZNep’s role on mice airway smooth muscle cells and human basal-like breast cancer cells, the NF-κB nuclear translocation was promoted via the IκB phosphorylation, which credited to the upregulated transcription of Foxc1 after the inhibition of EZH2 and subsequent H3K27me3 [12, 13]. Hence, we carried out the silence experiment of EZH2, and the Foxc1 inhibition experiment under DZNep treatment, and the data demonstrated that p-IKKα/β, p-IκBα were partly promoted through the DZNep-EZH2-H3K27me3-Foxc1 axis, which explained the activation and subsequent nuclear translocation of p-p65.

Furthermore, lots of factors regulated by osteoclasts, like Wnt10b, sema3a, smad1/5/8, and so on, had been widely demonstrated to mediate the differentiation and function of osteoblasts [21, 41, 42]. Although the p-p65 nuclear translocation in the osteoblast was reported to reduce osteogenesis by inhibiting the phosphorylation of Smad1/5, which promotes the osteoblast formation by inducing Runx2 expression [43], our data of DZNep on enhancing osteoblast formation were in agreement with the study that differentiation of MSC cell was shifted from adipocyte cell to osteoblasts due to the activation of Wnt gene transcription (Wnt1, Wnt6, and Wnt10a) [21]. In addition, similar to the reported EZH2-H3K27me3-Wnt1, Wnt6, Wnt10a axis, the Wnt4 had also been demonstrated transcriptionally regulating by H3K27me3 [44, 45]. Meanwhile, the Wnt4, which has been identified to promote osteogenesis due to the stimulated transcription of Runx2 caused by upregulated nuclear translocation of β-catenin and the expression of JNK and p38 [46], was also upregulated after the stimulation of DZNep in our research. Furthermore, the silence experiment of EZH2, and the Wnt4 inhibition experiment under DZNep treatment, respectively, demonstrated the vital role of EZH2 and Wnt4 during osteogenesis. All in all, we illustrated that the EZH2-H3K27me3-Wnt4 axis was partly contributed to the enhanced osteogenesis effect of DZNep.

Corresponding to the pro-osteoclastogenic and pro-osteogenic properties in vitro, our animal experiments in vivo further identified the enhanced role of DZNep on the healing of bone defect mice model. In our study, our bone defect mice with an intraperitoneal injection of DZNep showed markedly increased bone volume/tissue volume (BV/TV), trabecular thickness (Tb.Th) by the micro-CT analysis and H&E staining, when compared with the control group. Furthermore, our IHC staining indicated that the DZNep administration did increase the number of TRAP + Multinucleated cells and the immunofluorescence staining explicitly displayed the enhanced OCN expression on the treatment group than the control group. It means that DZNep could promote mice bone defect healing by stimulating osteoclastogenesis and osteogenesis at the same time.

However, as the earliest exploration study of the DZNep effect on osteoclasts and bone defect, our study still has several weaknesses. Firstly, further exploration of the underlying intermolecular regulation between osteoclast and osteoblast after DZNep treatment is still needed, which could better demonstrate how and why increased osteoclastic differentiation and activity at early phases can influence osteogenesis and be beneficial for bone defect closure. Secondly, it needs to be concerned that whether pharmacological therapy can fill any bone defect, such as critical-size defect. Larger size of the defect should be conducted in future experiment and the effect from pharmacological therapy in this regard should be identified. Thirdly, it is not suitable to treat patients with bone defects and fractures before further effects in other bone sites of general body and other animal models, like the bone fracture mice model and so on, shall have evaluated.

## Conclusions

Our study confirmed that DZNep effectively promotes RANKL-induced osteoclastogenesis partly through the EZH2-H3K27me3-Foxc1 axis and subsequent activation of the canonical NF-κB signaling pathway, and dependents on the EZH2-H3K27me3-Wnt4 axis to some extent to significantly enhanced osteogenesis. We also demonstrated that osteogenesis and osteoclastogenesis were both facilitated by DZNep in vitro and in vivo. In a whole, the results of this study not only indicate the great potential therapeutic effect of DZNep on bone defect, but also point out that the EZH2, in addition to an anti-tumor target, might be a momentous target in bone defect treatment by affecting osteoclast and osteoblast simultaneously.

## Supplementary Information

**Additional file 1**. Supplementary Materials.

## Data Availability

All data and materials included in this study are available upon request by contact with the corresponding author.

## Abbreviations

EZH2: Enhancer of zeste homolog 2
H3K27me3: Trimethylamine the histone H3 lysine 27
DZNep: 3-Deazaneplanocin
BMMs: Bone marrow-derived macrophages
BMSC: Bone marrow-derived mesenchymal stem cells
NF-κB: Nuclear factor-κB
RANKL: Receptor activator of nuclear factor-κB ligand
MAPK: Mitogen-activated protein kinase
IKK: Inhibitor of nuclear factor kappa-B kinase
IκB: Inhibitor of nuclear factor kappa-B

## References

[1] Pan YM, Wang CG, Zhu M, Xing R, Cui JT, Li WM, Yu DD, Wang SB, Zhu W, Ye YJ (2016). STAT3 signaling drives EZH2 transcriptional activation and mediates poor prognosis in gastric cancer. Mol Cancer.

[2] Qu Y, Lu D, Jiang H, Chi X, Zhang H (2016). EZH2 is required for mouse oocyte meiotic maturation by interacting with and stabilizing spindle assembly checkpoint protein BubRI. Nucleic Acids Res.

[3] Chase A, Cross NC (2011). Aberrations of EZH2 in cancer. Clin Cancer Res.

[4] Compston JE, McClung MR, Leslie WD (2019). Osteoporosis. Lancet.

[5] Langdahl B, Ferrari S, Dempster DW (2016). Bone modeling and remodeling: potential as therapeutic targets for the treatment of osteoporosis. Ther Adv Musculoskelet Dis.

[6] Yahara Y, Barrientos T, Tang YJ, Puviindran V, Nadesan P, Zhang H, Gibson JR, Gregory SG, Diao Y, Xiang Y (2020). Erythromyeloid progenitors give rise to a population of osteoclasts that contribute to bone homeostasis and repair. Nat Cell Biol.

[7] Hankenson KD, Gagne K, Shaughnessy M (2015). Extracellular signaling molecules to promote fracture healing and bone regeneration. Adv Drug Deliv Rev.

[8] Kylmaoja E, Nakamura M, Tuukkanen J (2016). Osteoclasts and remodeling based bone formation. Curr Stem Cell Res Ther.

[9] Fang C, Qiao Y, Mun SH, Lee MJ, Murata K, Bae S, Zhao B, Park-Min KH, Ivashkiv LB (2016). Cutting edge: EZH2 promotes osteoclastogenesis by epigenetic silencing of the negative regulator IRF8. J Immunol.

[10] Adamik J, Pulugulla SH, Zhang P, Sun Q, Lontos K, Macar DA, Auron PE, Galson DL (2020). EZH2 supports osteoclast differentiation and bone resorption via epigenetic and cytoplasmic targets. J Bone Miner Res.

[11] Zheng XJ, Li W, Yi J, Liu JY, Ren LW, Zhu XM, Liu SW, Wang JH, Du GH (2020). EZH2 regulates expression of FOXC1 by mediating H3K27me3 in breast cancers. Acta Pharmacol Sin.

[12] Zhu J, Wang W, Wu X (2020). Isorhynchophylline exerts anti-asthma effects in mice by inhibiting the proliferation of airway smooth muscle cells: the involvement of miR-200a-mediated FOXC1/NF-κB pathway. Biochem Biophys Res Commun.

[13] Wang J, Ray PS, Sim MS, Zhou XZ, Lu KP, Lee AV, Lin X, Bagaria SP, Giuliano AE, Cui X (2012). FOXC1 regulates the functions of human basal-like breast cancer cells by activating NF-κB signaling. Oncogene.

[14] Liu Y, Miao Y, Gao X, Wang YY, Wang H, Zheng YW, Zhao ZY (2018). MicroRNA-200a affects the proliferation of airway smooth muscle cells and airway remodeling by targeting FOXC1 via the PI3K/AKT signaling pathway in ovalbumin-induced asthmatic mice. Cell Physiol Biochem.

[15] Dankbar B, Fennen M, Brunert D, Hayer S, Frank S, Wehmeyer C, Beckmann D, Paruzel P, Bertrand J, Redlich K (2015). Myostatin is a direct regulator of osteoclast differentiation and its inhibition reduces inflammatory joint destruction in mice. Nat Med.

[16] Tanos T, Marinissen MJ, Leskow FC, Hochbaum D, Martinetto H, Gutkind JS, Coso OA (2005). Phosphorylation of c-Fos by members of the p38 MAPK family. Role in the AP-1 response to UV light. J Biol Chem.

[17] Iotsova V, Caamaño J, Loy J, Yang Y, Lewin A, Bravo R (1997). Osteopetrosis in mice lacking NF-kappaB1 and NF-kappaB2. Nat Med.

[18] Hemming S, Cakouros D, Codrington J, Vandyke K, Arthur A, Zannettino A, Gronthos S (2017). EZH2 deletion in early mesenchyme compromises postnatal bone microarchitecture and structural integrity and accelerates remodeling. FASEB J.

[19] Krämer M, Dees C, Huang J, Schlottmann I, Palumbo-Zerr K, Zerr P, Gelse K, Beyer C, Distler A, Marquez VE (2013). Inhibition of H3K27 histone trimethylation activates fibroblasts and induces fibrosis. Ann Rheum Dis.

[20] Kemp CD, Rao M, Xi S, Inchauste S, Mani H, Fetsch P, Filie A, Zhang M, Hong JA, Walker RL (2012). Polycomb repressor complex-2 is a novel target for mesothelioma therapy. Clin Cancer Res.

[21] Jing H, Liao L, An Y, Su X, Liu S, Shuai Y, Zhang X, Jin Y (2016). Suppression of EZH2 prevents the shift of osteoporotic MSC fate to adipocyte and enhances bone formation during osteoporosis. Mol Ther.

[22] Zhang P, Yang X, Ma X, Ingram DR, Lazar AJ, Torres KE, Pollock RE (2015). Antitumor effects of pharmacological EZH2 inhibition on malignant peripheral nerve sheath tumor through the miR-30a and KPNB1 pathway. Mol Cancer.

[23] Gannon OM, Merida de Long L, Endo-Munoz L, Hazar-Rethinam M, Saunders NA (2013). Dysregulation of the repressive H3K27 trimethylation mark in head and neck squamous cell carcinoma contributes to dysregulated squamous differentiation. Clin Cancer Res.

[24] Cheng LL, Itahana Y, Lei ZD, Chia NY, Wu Y, Yu Y, Zhang SL, Thike AA, Pandey A, Rozen S (2012). TP53 genomic status regulates sensitivity of gastric cancer cells to the histone methylation inhibitor 3-deazaneplanocin A (DZNep). Clin Cancer Res.

[25] Fiskus W, Wang Y, Sreekumar A, Buckley KM, Shi H, Jillella A, Ustun C, Rao R, Fernandez P, Chen J (2009). Combined epigenetic therapy with the histone methyltransferase EZH2 inhibitor 3-deazaneplanocin A and the histone deacetylase inhibitor panobinostat against human AML cells. Blood.

[26] Crea F, Hurt EM, Mathews LA, Cabarcas SM, Sun L, Marquez VE, Danesi R, Farrar WL (2011). Pharmacologic disruption of Polycomb Repressive Complex 2 inhibits tumorigenicity and tumor progression in prostate cancer. Mol Cancer.

[27] Kim BS, Yang SS, Lee J (2017). Precoating of biphasic calcium phosphate bone substitute with atelocollagen enhances bone regeneration through stimulation of osteoclast activation and angiogenesis. J Biomed Mater Res A.

[28] Jiang M, Liu R, Liu L, Kot A, Liu X, Xiao W, Jia J, Li Y, Lam KS, Yao W (2020). Identification of osteogenic progenitor cell-targeted peptides that augment bone formation. Nat Commun.

[29] Chen X, Chen X, Zhou Z, Mao Y, Wang Y, Mayo Z, Xuyo W, Qin A, Zhang S (2019). Nirogacestat suppresses RANKL-Induced osteoclast formation in vitro and attenuates LPS-Induced bone resorption in vivo. Exp Cell Res.

[30] Touaitahuata H, Morel A, Urbach S, Mateos-Langerak J, de Rossi S, Blangy A (2016). Tensin 3 is a new partner of Dock5 that controls osteoclast podosome organization and activity. J Cell Sci.

[31] Chen YC, Sosnoski DM, Mastro AM (2010). Breast cancer metastasis to the bone: mechanisms of bone loss. Breast Cancer Res.

[32] Debnath S, Yallowitz AR, McCormick J, Lalani S, Zhang T, Xu R, Li N, Liu Y, Yang YS, Eiseman M (2018). Discovery of a periosteal stem cell mediating intramembranous bone formation. Nature.

[33] Liu Z, Mar KB, Hanners NW, Perelman SS, Kanchwala M, Xing C, Schoggins JW, Alto NM (2019). A NIK-SIX signalling axis controls inflammation by targeted silencing of non-canonical NF-κB. Nature.

[34] Oryan A, Alidadi S (2018). Reconstruction of radial bone defect in rat by calcium silicate biomaterials. Life Sci.

[35] Srouji S, Blumenfeld I, Rachmiel A, Livne E (2004). Bone defect repair in rat tibia by TGF-beta1 and IGF-1 released from hydrogel scaffold. Cell Tissue Bank.

[36] Vannucci L, Brandi ML (2016). Healing of the bone with anti-fracture drugs. Expert Opin Pharmacother.

[37] Yan J, Yin Y, Zhong W, Wang C, Wang Z (2015). CD137 regulates NFATc1 expression in mouse VSMCs through TRAF6/NF-κB p65 signaling pathway. Mediators Inflamm.

[38] Takayanagi H (2007). Osteoimmunology: shared mechanisms and crosstalk between the immune and bone systems. Nat Rev Immunol.

[39] Mincheva S, Garcera A, Gou-Fabregas M, Encinas M, Dolcet X, Soler RM (2011). The canonical nuclear factor-κB pathway regulates cell survival in a developmental model of spinal cord motoneurons. J Neurosci.

[40] Perkins ND (2007). Integrating cell-signalling pathways with NF-kappaB and IKK function. Nat Rev Mol Cell Biol.

[41] Sonoda S, Murata S, Nishida K, Kato H, Uehara N, Kyumoto YN, Yamaza H, Takahashi I, Kukita T, Yamaza T (2020). Extracellular vesicles from deciduous pulp stem cells recover bone loss by regulating telomerase activity in an osteoporosis mouse model. Stem Cell Res Ther.

[42] Iezaki T, Onishi Y, Ozaki K, Fukasawa K, Takahata Y, Nakamura Y, Fujikawa K, Takarada T, Yoneda Y, Yamashita Y (2016). The transcriptional modulator interferon-related developmental regulator 1 in osteoblasts suppresses bone formation and promotes bone resorption. J Bone Miner Res.

[43] Xie Z, Yu H, Sun X, Tang P, Jie Z, Chen S, Wang J, Qin A, Fan S (2018). A novel diterpenoid suppresses osteoclastogenesis and promotes osteogenesis by inhibiting Ifrd1-mediated and IκBα-mediated p65 nuclear translocation. J Bone Miner Res.

[44] Garcia-Moreno SA, Lin YT, Futtner CR, Salamone IM, Capel B, Maatouk DM (2019). CBX2 is required to stabilize the testis pathway by repressing Wnt signaling. PLoS Genet.

[45] Katoh N, Kuroda K, Tomikawa J, Ogata-Kawata H, Ozaki R, Ochiai A, Kitade M, Takeda S, Nakabayashi K, Hata K (2018). Reciprocal changes of H3K27ac and H3K27me3 at the promoter regions of the critical genes for endometrial decidualization. Epigenomics.

[46] Li X, Li Z, Wang J, Li Z, Cui H, Dai G, Chen S, Zhang M, Zheng Z, Zhan Z (2019). Wnt4 signaling mediates protective effects of melatonin on new bone formation in an inflammatory environment. FASEB J.

