# Supplementary material for: DZNep promotes mouse bone defect healing via enhancing both osteogenesis and osteoclastogenesis
Source: Stem Cell Res Ther. 2021 Dec 20;12:605. doi: 10.1186/s13287-021-02670-6 (PMC8686256; doi:10.1186/s13287-021-02670-6)
Supplement: Supplementary file 1 — Additional file 1. Supplementary Materials. [file 13287_2021_2670_MOESM1_ESM.docx]

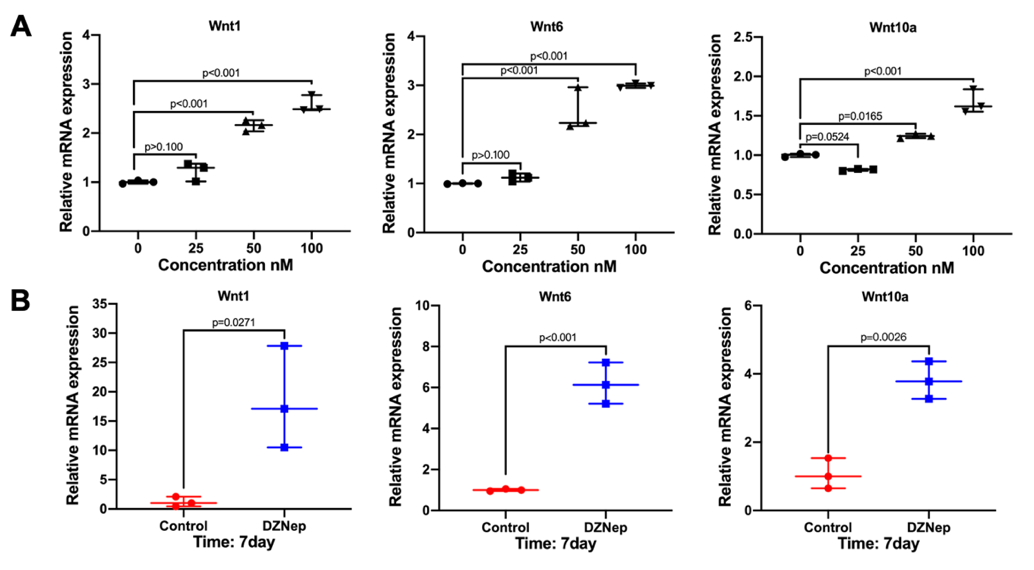


Supplement Figure 1. DZNep promoted the expression of wnt signaling pathway-specific genes Wnt1, Wnt6, Wnt10a in osteoblasts. (A) Expression of the wnt signaling pathway-specific genes Wnt1, Wnt6, and Wnt10a in BMSC treated with 0, 25, 50, 100 nM of DZNep combined with osteoblast cultured medium for 24 hours. (B) Expression of the wnt signaling pathway-specific genes Wnt1, Wnt6, and Wnt10a in BMSC treated with or without 100 nM of DZNep combined with osteoblast cultured medium for 7 days. Gene expression was analyzed by real-time PCR. mRNA expression levels were normalized relative to the expression of β-actin. (**p* < 0.05; ***p* < 0.01; ****p* <0.001; *****p* <0.0001). Data are expressed as median and interquartile range，n=3.


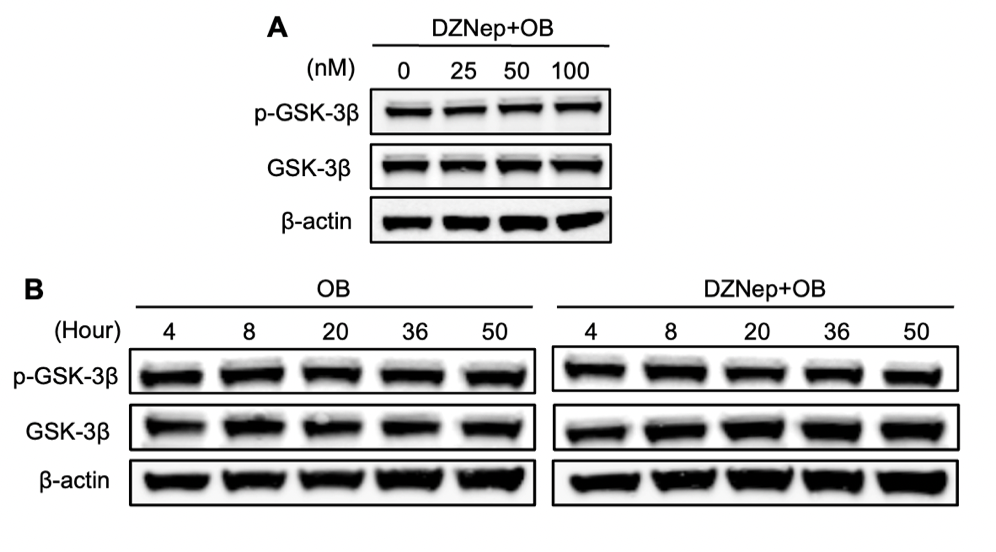


**Supplement figure 2.** DZNep enhances osteogenesis independent of the phosphorylation of p-GSK-3β. (A) GSK-3βand p-GSK-3β expression levels in BMSC treated with 0, 25, 50, 100 nM of DZNep combined with osteoblast cultured medium for 24 hours. (B) GSK-3β and p-GSK-3β expression levels in BMSC treated with 100 nM DZNep and osteoblast cultured medium for 4, 8, 20, 36, 50 hours.

**
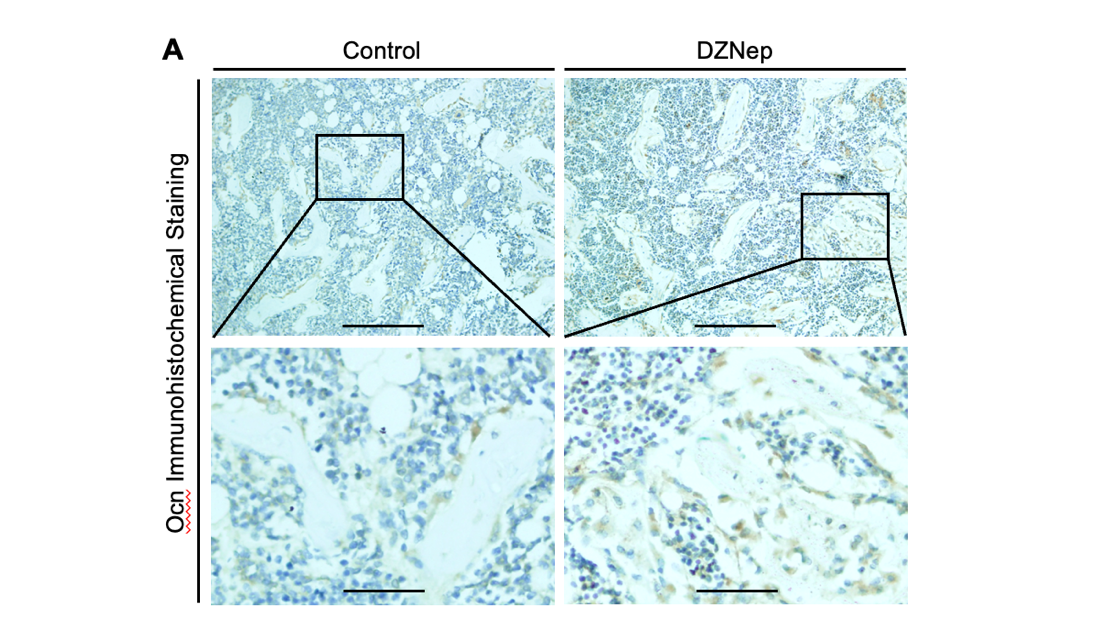
**

**Supplement Figure 3.** DZNep promoted the expression of Ocn in vivo. (A) Immunohistochemical (magnification × 20 and × 80) staining, respectively, of Ocn in the defect tibiae sections. Scale bar = 10 μm and 2.5 μm.
